# Changes in Chemical Composition of Lentils, Including Gamma-Aminobutyric Acid and Volatile Compound Formation during Submerged and Solid-State Fermentation with *Pediococcus acidilactici*

**DOI:** 10.3390/foods13081249

**Published:** 2024-04-19

**Authors:** Ernestas Mockus, Vytaute Starkute, Dovile Klupsaite, Vadims Bartkevics, Anastasija Borisova, Lina Sarunaite, Ausra Arlauskiene, João Miguel Rocha, Elena Bartkiene

**Affiliations:** 1 Institute of Animal Rearing Technologies, Lithuanian University of Health Sciences, Tilzes Str. 18, LT-47181 Kaunas, Lithuania; ernestas.mockus@lsmu.lt (E.M.); vytaute.starkute@lsmu.lt (V.S.); dovile.klupsaite@lsmu.lt (D.K.); 2 Department of Food Safety and Quality, Lithuanian University of Health Sciences, Tilzes Str. 18, LT-47181 Kaunas, Lithuania; 3 Institute of Food Safety, Animal Health and Environment BIOR, Lejupes iela 3, LV-1076 Riga, Latvia; vadims.bartkevics@bior.lv (V.B.); anastasija.borisova@bior.lv (A.B.); 4 Lithuanian Research Centre for Agriculture and Forestry, Institute of Agriculture Instituto 1, Akademija, LT-58344 Kėdainiai, Lithuania; lina.sarunaite@lammc.lt (L.S.); ausra.arlauskiene@lammc.lt (A.A.); 5 CBQF—Centro de Biotecnologia e Química Fina—Laboratório Associado, Escola Superior de Biotecnologia, Universidade Católica Portuguesa, Rua Diogo Botelho 1327, 4169-005 Porto, Portugal; jmfrocha@fc.up.pt; 6 LEPABE—Laboratory for Process Engineering, Environment, Biotechnology and Energy, Faculty of Engineering, University of Porto, Rua Dr. Roberto Frias, 4200-465 Porto, Portugal; 7 ALiCE—Associate Laboratory in Chemical Engineering, Faculty of Engineering, University of Porto, Rua Dr. Roberto Frias, 4200-465 Porto, Portugal

**Keywords:** amino acids, biogenic amines, fatty acids, fermentation, gamma-aminobutyric acid, trace elements, volatile compounds, lentils

## Abstract

The aim of this study was to evaluate and compare the characteristics of non-treated and fermented [via submerged (SMF) and solid-state (SSF) fermentation using *Pediococcus acidilactici*] lentils (*Lens culinaris*) grown either in pure stands (L) or relay intercropped with winter rye (LR). It was observed that the lentils were suitable substrate for lacto-fermentation. Most of the free amino acid concentrations increased in lentils after both fermentations. The highest concentration of γ-aminobutyric acid was found in SSF LR samples. However, fermentation led to higher biogenic amines (BA) content in lentils. The most abundant fatty acid in lentils was C18:2. SSF lentils showed more complex volatile compound (VC) profiles (with between nine and seventeen new VCs formed), whereas, in SMF samples, between two and five newly VCs were formed. When comparing lentil grown types, L contained significantly higher concentrations of Na, K, Ca, P, Mn, and Se, while LR contained significantly higher concentrations of Fe and Ni. To sum up, fermentation with lactic acid bacteria (LAB) contributed to the improved biological value of lentils; still, the quantity of BA needs to be considered. Further investigations into the *P. acidilactici* metabolism of certain compounds (such as phenolic and antinutritional compounds) in lentils during fermentation ought to be carried out.

## 1. Introduction

Cultivated lentil (*Lens culinaris*), one of the ancient crops, is highly important due to its high protein content (20–25%), diverse and rich profile of essential microelements (iron, zinc, copper, manganese, and phosphorus), and significant amounts of B group vitamins [thiamine (B1), riboflavin (B2), niacin (B3), pantothenic acid (B5), pyridoxine (B6), and folic acid (B9)] [1]. Lentil landraces contain a diverse profile of carbohydrates, which consists of slowly digestible starch, prebiotic dietary fibre (such as raffinose family oligosaccharides and fructooligosaccharides), and sugar alcohols [2,3]. Moreover, leguminous crops contain various phytochemicals with beneficial properties, such as the ability to promote or inhibit enzymatic reactions, provide antioxidant properties, and inhibit harmful gut bacteria [4,5]. 

The popularity of lentil crops in the food and feed industries is increasing, as the worldwide production rate has grown two-fold, up to 6.54 million metric tons in 2020 [3]. Although lentils are not as popular in Europe as in America, our traditions and older research show that the cultivation of lentils in Lithuania could be advanced: we could breed more suitable varieties and apply sustainable farming technologies to their cultivation [6]. Researchers are investigating various mixed cropping systems of legume with cereals by improving the cycling and uptake of nutrients. Mixed cropping demonstrates a yield advantage in different climatic conditions, confirming the suitability of cereal and legume intercropping in North and Central Europe [7]. Its potential mechanisms and effects consist of competition (niche differentiation, resource use sharing, and weed control), diversity (pest and disease control), facilitation (physical support and the excretion of N and allelochemicals), and associated diversity (habitats for natural predators; litter diversity enhances soil microbial diversity) [8]. The mixed cropping technique is intended to provide long soil cover with living cover crop and its maintenance after the main crop is harvested may protect the soil against erosion, provide the next crop with nitrogen, prevent nitrate leaching, and improve plant nutrition and production quality [9,10].

However, its digestive properties are limited, due to various antinutritional factors [11,12]. Fermentation is often described as classic, simple, and low-cost bioprocessing method that helps to increase a product’s shelf-life and improve its nutritional and organoleptic properties by reducing undesirable compounds and supplementing the food matrix with essential amino acids and vitamins [13,14]. Moreover, fermentation can reduce various antinutritional factors, such trypsin inhibitors, tannins, and phytic acid [13] and can improve the food matrix’s digestibility by promoting the hydrolysis of proteins [15].

The fermentation of various matrices can be performed under submerged (SMF) and solid-state fermentation (SSF) conditions [16,17]. On one hand, SMF is defined as a type of process wherein the anaerobic or partially anaerobic fermentation of a substrate is performed in the presence of free water, also known as a liquid medium [18]. On the other hand, SSF is defined as a type of process wherein fermentation occurs on a solid matrix in the absence of free water [18,19]. The SMF process is fairly popular in the industry for producing various fermentation products, due to its ease of control over the fermentation process, shorter fermentation duration, consistent productivity, and the ease of the purification of the products [18,19]. Nevertheless, SSF has advantages over SMF, such as a higher productivity, lower energy expenditures, simpler instrumentation, lower catabolic repression, a lower risk of contamination, and the better microbial growth and production of secondary metabolites [18,19,20,21].

Although the fermentation of legumes provides numerous benefits to their nutritional profile, it should be emphasized that fermentation is often associated with the formation of exogenous biogenic amines (BAs) [22,23]. Although BAs are essential to some biological functions, such as the synthesis of hormones or alkaloids, the excess consumption of exogenous BA can lead to various food poisoning symptoms, such as headaches, vomiting, diarrhoea, dizziness, nausea, or pseudo-allergic reactions [24,25]. For this reason, BA control in fermented products is of the utmost importance.

Previous studies have reported changes in nutritional profile, phenolic compounds, antioxidant properties, and the antidiabetic potential of lentils during fungal solid-state fermentation [26,27,28,29]. It was also reported that the phenolic content, protein quality, and digestibility of lentil proteins were improved using water kefir seed fermentation [30]. 

Several studies have been conducted on lentil fermentation with lactic acid bacteria (LAB). The study of Chen et al. [31] revealed that the solid-state co-fermentation of lentils using LAB and *Bacillus subtilis* natto increased the antioxidant activity and LAB proliferation in the fermented lentil products. Torino et al. [14] used liquid and solid-state fermentations with *Lactobacillus plantarum* and *Bacillus subtilis* to produce water-soluble fermented lentil extracts with antioxidant and antihypertensive properties. The study of De Pasquale et al. [32] on the lactic acid fermentation of gelatinized yellow and red lentil flour was carried out in order to analyse the alterations in total phenolic compounds, protein digestibility, antinutritional factors (tannins, trypsin inhibitor activity, phytic acid content, etc.), and antioxidant capacity. Another study was carried out to examine the impact of sonication or precooking and fermentation with *Lactobacillus plantarum* and *Pediococcus acidilactici* on the antinutritional factors of several legume flours, including lentil [13]. Budryn et al. [33] examined the impact of fermentation with *Lactobacillus casei* on increasing the content of isoflavonoids and microbiological safety in lentils. The above-mentioned previous studies on lentil fermentation varied regarding the type of initial lentil raw material used, the microorganisms used, and the examined characteristics, mainly antioxidant and antinutritional, of the fermented product. Therefore, to the best of our knowledge, this study is the first to use the differently grown lentil variety ‘Danaja’ for fermentation with a newly isolated *Pediococcus acidilactici* strain, in order to compare the changes in compounds such as biogenic amines, volatile compounds, fatty acids, and γ-aminobutyric acid in the obtained products.

The aim of this study was to analyse and compare the physical, chemical, and microbiological characteristics [chromaticity parameters, pH, BAs, volatile compounds (VCs), fatty (FA) and free amino (FAA) acids, γ-aminobutyric acid (GABA) concentration, and lactic acid bacteria (LAB) viable count] of non-treated and fermented (via SMF and SSF with *Pediococcus acidilactici* strain) lentils grown either in pure stands (L) or relay intercropped with winter rye (LR).

## 2. Materials and Methods

### 2.1. Lentil and Lactic Acid Bacteria Strains Used for Fermentation

The grains of lentil variety ‘Danaja’ (*Lens culinaris* Medik.) were provided by the Lithuanian Research Centre for Agriculture and Forestry (LAMMC, Akademija, Kėdainiai distr., Lithuania). Field trials were conducted during the 2022 vegetation period at the experimental base of the LAMMC (55°39′ N 23°57′ E). The field experiment was designed in four replications (plot size 3 × 20 m), which were randomly arranged. The trial was conducted under organic growing conditions. The soil of the experimental site was a loamy *Endocalcaric Epigleyic Cambisol* (Drainic, Loamic) CM-can.glp-dr.lo. The characteristics of the soil arable layer (0–25 cm) were as follows: pH 7.2, humus content 2.7%, total nitrogen 117 mg/kg, available phosphorus (P_2_O_5_) 51 mg/kg, and available potassium (K_2_O) 68 mg/kg. Lentil was grown either in pure stands (L) or was relay intercropped with winter rye (LR), which involves growing a second crop after harvesting the first (see supplementary file: Figure S1). Both crops were planted in May in the same plot. The lentils were harvested in September. Winter rye was the cover crop between the lentil rows in the sowing year and was left over the winter period as a cereal for grain harvest.

**Figure 1:**
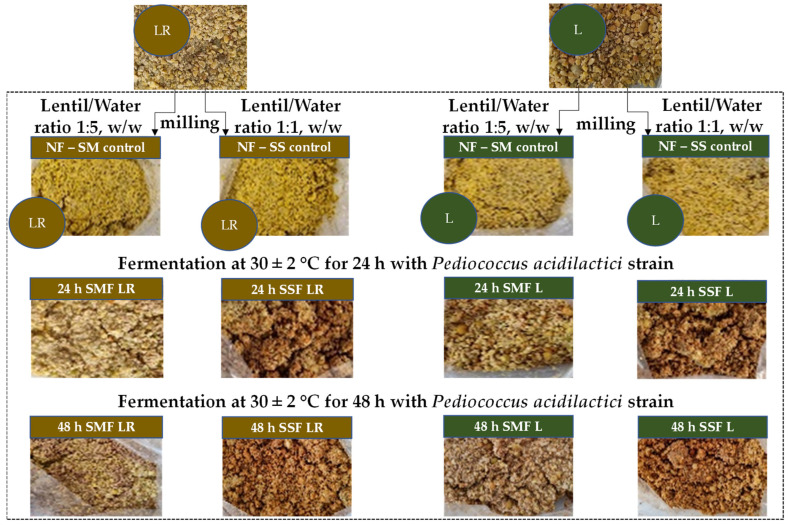
The principal scheme of the experiment (L—lentil obtained from pure stands; LR—lentil obtained from relay incorporated with winter rye; NF—non-fermented; SM—submerged conditions, SS—solid-state conditions; SMF—submerged fermented; SSF—solid-state fermented).

The lentils (moisture content 14%) were milled with a Laboratory Mill 120 (Perten Instruments AB, Stockholm, Sweden) until the particle size was 1–2 mm and were then mixed with water: for submerged (SM) conditions, the lentil/water ratio was 1:5 (weight/weight, *w*/*w*), and, for solid-state (SS) conditions, the lentil/water ratio was 1:1 (*w*/*w*). In total, the following twelve sample groups were obtained: four control groups—LR and L control groups for SM and SS conditions (C_LR_SM, C_LR_SS, C_L_SM, and C_L_SS); four groups fermented for 24 h under SM and SS conditions (SMF_LR_24, SSF_LR_24, SMF_L_24, and SSF_L_24); and four groups fermented for 48 h under SM and SS conditions (SMF_LR_48, SSF_LR_48, SMF_L_48, and SSF_L_48). The principal scheme of the experiment is given in Figure 1.

The *Pediococcus acidilactici* strain, previously isolated from spontaneous fermented cereal, was used for lentil fermentation [34]. Previous studies disclosed that the *Pediococcus acidilactici* strain is able to ferment various carbohydrate- and protein-rich matrices and multiply rapidly, reaching 3.20 ± 0.1 log_10_ CFU/mL within 2 h [34,35].

Before the experiments, the *Pediococcus acidilactici* strain was stored at −80 °C (Microbank system, Pro-Lab Diagnostics, Birkenhead, UK) and multiplied in de Man–Rogosa–Sharpe (MRS) broth (Oxoid Ltd., Hampshire, UK) at 30 ± 2 °C for 24 h, before using it for lentil fermentation. Milled lentil, water, and a suspension of *Pediococcus acidilactici* strain [3% of dry matter (d.m.) of the lentil mass, containing 8.7 log10 CFU/mL] was fermented at 30 ± 2 °C for 24 and 48 h in a chamber incubator (Memmert GmbH + Co. KG, Schwabach, Germany). For 100 g of lentils, 500 mL and 100 mL of water was added for submerged fermentation and solid-state fermentation, respectively. Non-fermented samples (mixed with water) were analysed as a control.

### 2.2. Evaluation of Acidity and the Microbiological and Chromaticity Characteristics of Lentil Wholemeal Samples

The pH of the lentil samples was measured using a pH electrode (PP-15; Sartorius, Goettingen, Germany). The LAB viable counts were determined according to the method described by Bartkiene et al. [36]. The chromaticity characteristics of lentil samples were evaluated on the samples’ surfaces using a CIE L*a*b* system (CromaMeter CR-400, Konica Minolta, Tokyo, Japan). The results were expressed as the CIE colour values L* (brightness/darkness), a* (redness/greenness), and b* (yellowness/blueness).

### 2.3. Analysis of Free Amino Acids in Lentil Wholemeal Samples

Sample preparation and dansylation were performed according to the method of Hua-Lin Cai et al., with some modifications [37]. A homogenous sample (~1 g) was weighed into a 50 mL tube, and analytes were extracted with 10 mL of aqueous 0.1 M HCl solution, by blending the mixture with a laboratory blender and shaking for 30 min. The resultant mixture was centrifuged at 4000 rpm for 10 min. For derivatization, 100 µL of resultant supernatant was diluted to 500 µL with 0.1 M HCl solution. The resultant mixture was made alkaline by adding 40 µL of 2 M NaOH and 70 µL of saturated NaHCO_3_ solution. Derivatization was performed by adding 0.5 mL of 20 mg/mL dansyl chloride solution in acetonitrile and heating the resulting mixture at 60 °C for 30 min. The reaction mixture was cooled down to room temperature, centrifuged at 12 k rotations per minute (rpm) for 5 min, at room temperature, and filtered with a 0.20 µm membrane filter (Chromafil^®^Xtra PA-20/25, nylon, Düren, Germany) into the auto-sampler vial. The concentration of analytes was determined using the Varian ProStar high-performance liquid chromatography (HPLC) system (Varian Corp., Palo Alto, CA, USA), which included two ProStar 210 pumps, a ProStar 410 autosampler, and a Thermo Scientific LCQ Fleet Ion trap mass detector (Thermofisher Scientific, San Jose, CA, USA). For analyte detection, the mass spectrometer was operated in the positive ionisation consecutive reaction monitoring mode, for specific ions which corresponded to derivatized analyte. The concentration of analyte was determined from the calibration curve, which was obtained by derivatizing analytes at different concentrations. For the separation of derivatives, a Discovery^®^ HS C18 column (150 mm × 4.6 mm-ϕ, 5 µm-ϕ particle size; SupelcoTM Analytical, Bellefonte, PA, USA) was used. The mobile phase A was 0.1% formic acid in 5% aqueous acetonitrile and phase B was 0.1% in acetonitrile. A 10 µL injection volume was used for analyses. The analytical gradient was as follows: 0 to 1.0 min, 15 to 50% B, (linear gradient, flow rate 1 mL/min); 1.0 to 3.0 min (flow-rate 1 mL/min); 3.0 to 3.5 min (flow-rate 1.0 to 0.3 mL/min); 3.5 to 10 min (linear gradient), 50 to 70% B; 10 to 15.0 min (linear gradient), 70 to 90% B; 15 to 30 min, 90 to 95% B; and followed by column re-equilibration for 11 min with 15% B (flow-rate increased to 0.8 mL/min).

### 2.4. Determination of the Biogenic Amines Content

The extraction and determination of BAs in non-fermented and fermented lentil samples followed the procedures developed by Ben-Gigirey et al. [38]. Perchloric acid (0.4 mol/L, 10 mL) containing a known amount of 1,7-diaminoheptane, as an internal standard (ISTD), was added to 3 g of sample, and the mixture was homogenized with Ultra-Turrax apparatus (IKA Labortechnik, Staufen, Germany) and centrifuged at 3000× *g* for 10 min at room temperature. The residue was extracted again with an equal volume of 0.4 mol/L perchloric acid. Both supernatants were combined, and the final volume was adjusted to 30 mL with 0.4 mol/L perchloric acid. The extract was filtered through a Whatman No. 1 paper filter. One millilitre of extract or standard solution was mixed with 200 mL of 2 mol/L NaOH solution and 300 mL of saturated NaHCO_3_ solution. Two millilitres of 10 mg/mL solution of 5-(dimethylamino)naphthalene-1-sulfonyl chloride (dansyl chloride) in acetone was added to the mixture and incubated at 40 °C for 45 min. Residual dansyl chloride was removed by the addition of 100 mL of 25 mg/L aqueous ammonia solution. After incubation at room temperature for 30 min, the mixture was adjusted to 5 mL with acetonitrile. Finally, the mixture was centrifuged at 3000× *g* for 5 min at room temperature; the supernatant was then filtered through a 0.2 μm filter (Millipore, nylon, Bedford, MA, USA) and stored at 25 °C until HPLC analysis. An Agilent 1200 HPLC system (Carlsbad, CA, USA) equipped with a diode array detector (DAD) and Chemstation liquid chromatography (LC) software (version B.04.03) was employed in combination with a Chromolith C_18_ HPLC column (diode array detector (DAD) 100 mm × 4.6 mm-ϕ × 4 μm-ϕ particle size, Merck KGaA/EMD Chemicals, Darmstadt, Germany). Ammonium acetate (0.1 mol/L) and acetonitrile were used as the mobile phases at the flow-rate of 0.45 mL/min. The injected sample volume was 10 μL and the biogenic amines were monitored at 254 nm wavelength (λ). The detection limits for standard amine solutions were approximately 0.1 mg/kg.

### 2.5. Fatty Acid Composition Analysis

The fatty acid composition of the lentil samples was determined using a GCMS-QP2010 (Shimadzu, Kyoto, Japan) gas chromatograph with a mass spectrometer (GC-MS). The fatty acid methyl ester (FAME) concentration was determined using a calibration curve, and the results were expressed as percentage of total FAME concentration in fat. Samples were prepared by extracting 1 g of the homogenized sample with 10 mL of extraction solution [chloroform–methanol, 2:1 (volume/volume, *v*/*v*)] by shaking for 1 h. Afterwards, the mixture was centrifuged at 4000 rpm for 5 min at room temperature, and 5 mL of the organic phase was washed with 1 mL of 0.9% aqueous sodium chloride solution. The resulting mixture was centrifuged at 4000 rpm for 5 min at room temperature, and 1 mL of organic phase was evaporated using nitrogen gas. The resulting residue was dissolved in 1 mL of hexane and reacted with 70 µL of methylation reagent (2 mol/L of KOH in methanol) by vortexing and shaking using a laboratory shaker for 30 min. The mixture was centrifuged at 13.2 k rpm for 5 min at room temperature and the upper layer was used for the analysis. We used a Capillary Stabilwax-MS column (30 m × 0.25 mm-ϕ internal diameter (ID) × 0.25 µm-ϕ film thickness). The mass spectrometer operated at full scan mode. The analyte was injected in split mode at a 1:60 split ratio. The following parameters were used: MS ion source temperature: 240 °C; MS interface temperature: 240 °C; helium (carrier gas) flow-rate: 0.90 mL/min; injector: 240 °C; oven temperature program: 50 °C (4 min), 10 °C min^−1^ to 110 °C (1 min), 15 °C min^−1^ to 160 °C (2 min), 2.5 °C min^−1^ to 195 °C (1 min), 2 °C min^−1^ to 230 °C (1 min), and 2 °C min^−1^ to 240 °C (12 min).

### 2.6. Evaluation of Volatile Compound Profiles

The VCs of samples were analysed using gas chromatography–mass spectrometry. A solid-phase microextraction (SPME) device with Stableflex™ fibre, coated with a 50 μm-ϕ PDMS-DVB-Carboxen™ layer (Supelco, USA), was used for analyses. For the headspace extraction of the samples, 1 g of sample and 12 mL of 1 M phosphate buffer (pH = 3) were transferred to the 20 mL extraction vial, mixed, sealed with a polytetrafluoroethylene septum, and thermostatted at 60 °C for 30 min before exposing the fibre in the headspace. The fibre was exposed to the headspace of the vial for 10 min and desorbed in an injector liner for 2 min (splitless injection mode). The prepared samples were analysed with a GCMS-QP2010 (Shimadzu, Japan) gas chromatograph and mass spectrometer. The following conditions were used for analysis: injector temperature 250 °C; ion source temperature 220 °C; and interface temperature 260 °C. Helium was used as a carrier gas at 0.65 mL/min flow-rate. For the separation of VCs, a low polarity Rxi^®^-5MS column (Restek, Centre County, PA, USA) (length 30 m, coating thickness 0.25 μm-ϕ, inner diameter of 0.25 mm-ϕ) was used. The temperature gradient was programmed from starting at 40 °C (3 min hold) to 220 °C (5 °C/min) up to 310 °C (15°/min) (6 min hold). The VCs were identified according to mass spectrum libraries (National Institute of Technology spectral library (NIST11) and Flavors and Fragrances of Natural and Synthetic Compounds 2 spectral library (FFNSC2)). For identification purposes, alkane mixes (C8–C20) were analysed to obtain the retention indices of unknown compounds.

### 2.7. Analysis of Micro- and Macro-Elements Using Inductively Coupled Plasma Mass Spectrometry (ICP-MS)

For macro- and micro-element analysis in lentil samples, an Agilent 7700x ICP-MS (Agilent Technologies, Tokyo, Japan) and a software Mass Hunter Work Station for inductively coupled plasma mass spectrometry (ICP-MS), version B.01.01 (Agilent Technologies, Japan) were used. The samples were milled and homogenised (final particle size ≤ 150 µm). For the analysis, the following chemicals were used: nitric acid (concentration ≥ 69.0%) for trace analysis (Sigma-Aldrich, Paris, France); hydrogen peroxide, 30% *w*/*w*, extra pure (Scharlau, Sentmenat, Spain); and multielement standard solution V for ICP-MS calibration (Sigma-Aldrich, France). An Agilent 7700x ICP-MS (Agilent Technologies, Tokyo, Japan), with the software Mass Hunter Work Station for ICP-MS, version B.01.01 (Agilent Technologies, Japan), was used for analyses. The sample preparation for ICP-MS analysis was carried out as follows: in a microwave vessel, we accurately weighed 0.3 g of the homogenized sample, 2 mL of deionized water, 8 mL of concentrated nitric acid, and 2 mL of concentrated hydrogen peroxide, which were added together, and we waited for 2–8 h for reaction stabilization, until the formation of bubbles was finished. The vessel was sealed and heated in the microwave system. The following thermal conditions were applied: a 150 °C temperature was reached in approximately 20 min and remained for 30 min and then, 200 °C was reached in approximately 20 min and maintained for 30 min for the completion of specific reactions. After cooling (approximately 40 min), the prepared solution was filtered through the filter with a pore size 8–10 μm-ϕ. The solution was transferred to a 50 mL volumetric flask and filled with deionized water to a 50 mL volume. The following operating conditions of the Agilent 7700x ICP-MS were used for the analysis of the samples: plasma mode—normal, robust; RF forward power 1300 W; sampling depth 8.00 mm; carrier gas-flow 0.6 L/min; dilution gas-flow 0.4 L/min; spray chamber temperature 2 °C; extraction lens 1 V; and kinetic energy discrimination 3 V.

### 2.8. Statistical Analysis

Microbiological and physicochemical results were expressed as the mean (n = 3) ± standard error (SE). The experiment was performed by preparing three parallel samples for fermentation, and one sample was taken from each for analysis. In order to evaluate the effects of the different factors (viz. growing conditions of lentil: pure stands and relay incorporated with winter rye; different fermentation conditions; and different durations of the fermentation), the data were analysed using multivariate analysis of variance (ANOVA). A Tukey HSD (honest significant difference) post hoc test was conducted to detect differences among sample groups. To determine the normality, the Shapiro–Wilk test was used; for homoskedasticity evaluation, the homoskedasticity test, using the SPSS Statistical Package (v15.0, SPSS, Chicago, IL, USA), was performed. The linear Pearson’s correlation coefficients were calculated using the statistical package SPSS for Windows (v15.0, SPSS, Chicago, IL, USA). Correlation strength interpretation was performed according to the method described by Evans (1996) [39]. The results were recognized as statistically significant at *p* ≤ 0.05. Heatmap visualization and analysis was carried out using the R statistical programming software package “ComplexHeatmap” (version 2.14.0). Bar plot visualization was carried out using the “tidyverse” package (version 2.0.0).

## 3. Results and Discussion

### 3.1. Acidity and Microbiological and Chromaticity Characteristics of Lentil Wholemeal Samples

The pH values, chromaticity characteristics, and LAB viable counts of lentil samples are presented in Table 1. Comparing the pH values of non-fermented LR and L sample groups (SM and SS), higher pH values were observed in LR sample groups. However, after 24 and 48 h of SMF, the opposite tendencies were established; i.e., L samples showed lower pH values than LR groups. SSF conditions were more effective in reducing the pH values of L samples; however, when comparing SSF L and LR sample groups, lower pH values were attained in L sample groups (after 24 and 48 h of SSF). In both sample groups (LR and L) under both conditions (SMF and SSF), the pH values decreased between 24 and 48 h of fermentation.

Despite the pH differences in non-fermented samples (between C_LR_SM and C_L_SM; C_LR_SS and C_L_SS), significant differences in LAB viable counts were found only between C_LR_SM and C_L_SM samples. Contrasting non-fermented samples (C_LR_SM, C_LR_SS, C_L_SM, and C_L_SS), LAB viable counts were, on average, 4.67 log_10_ CFU/g. In comparison, for LAB numbers in different lentil sample groups (LR and L) with the same treatment, significant differences were also inexistent, and, in both sample groups (SMF and SSF), higher LAB viable counts were detected after 48 h of fermentation, with an average value of 8.23 log_10_ CFU/g. The analysed factors (type of fermentation and fermentation duration) and the interaction of growing conditions * fermentation duration were significant (*p* = 0.005, *p* < 0.001, and *p* = 0.003, respectively) for the LAB viable counts in lentil samples (see supplementary file: Table S1). Also, a strong negative correlation (r = −0.886, *p* < 0.001) between lentil pH values and LAB numbers was found (see supplementary file: Table S2).

The fermentation of leguminous crops can provide probiotic benefits in foods [13]. Moreover, it was reported that SSF is more commonly used for various biological conversion of substrates, because of its higher efficiency [40]. Our study showed that all the analysed factors (type of fermentation, growing conditions, and fermentation duration) and their interactions were significant (*p* < 0.001) on the pH values of lentil samples (see supplementary file: Table S1). The results also showed that, in all of the fermented lentil samples, LAB viable counts were higher than 6.0 log_10_ CFU/g; such a number of desirable microorganisms may ensure the probiotic properties of foodstuffs, if the strains used for fermentation are probiotics (which is the case here).

The carbohydrate metabolism of *P. acidilactici* strain was studied in 47 different carbon sources, and this strain demonstrated fermentation activity for 20 out of 47 tested carbohydrates [34]. Additionally, the optimal growth temperature for this strain was 30 °C. This characteristic can explain the differences in the fermentation activity of the *P. acidilactici* strain reported in the study of Byanju et al. [13], who found that, for *P. acidilactici* (provided by Lallemand Animal Nutrition–North America, Milwaukee, WI, USA), the exponential growth in lentil flour was observed between 6 and 24 h in SMF conditions. In addition, the pH decreased significantly during the first 24 h of fermentation, and the microbial population attained the highest value during this time period [13].

Throughout fermentation, the low pH of the substrate can damage both the non-desirable and desirable microorganism’s cell wall and cell membrane [41]. LAB adaptation to low pH conditions depends on their phenotype characteristics and other conditions under which cells are exposed to stress [42]. The molecular mechanisms of LAB adaptation and habituation to stress may overlap to a certain degree, but they are not completely identical [34]. This may explain the different results reported in distinct LAB species for the fermentation effectiveness parameters. However, the pH values and the number of LAB are the main characteristics of fermentation efficiency. This study showed that *P. acidilactici* is a suitable strain for lentil fermentation; furthermore, a 48 h fermentation duration was shown to be most suitable for lentil fermentation with this particular LAB strain, because statistically significant lower pH and higher LAB viable counts after 48 h of SMF and SSF were obtained, when compared with 24 h fermented samples.

In comparison, chromaticity characteristics of the non-fermented lentils (LR samples: C_LR_SM and C_LR_SS) showed, respectively, on average, 1.03 and 10.6% lower brightness (L*) and yellowness (b*), as well as, on average, 42.3% higher redness (a*), in comparison with L samples. In all cases, the same tendencies were found: with an increasing duration of fermentation, the L* and b* values of the lentils decreased and the a* values increased. Lentil L*, a*, and b* values showed statistically significant correlations with pH and LAB viable counts (with pH: r = 0.763, *p* < 0.001; r = −0.679, *p* < 0.001; and r = 0.839, *p* < 0.001, respectively; with LAB viable counts: r = −0.710, *p* < 0.001; r = 0.552, *p* < 0.001; and r = −0.799, *p* < 0.001, respectively). Analysed factors (fermentation conditions, lentil growing conditions, and fermentation duration) and their interactions were statistically significant (*p* < 0.001) for the colour coordinate values of lentil samples, except for the interaction between growing conditions, type of fermentation, and fermentation durations on a* values (see supplementary file: Table S1).

The colour of the lentil is a key quality parameter due to its impact on the acceptability of the legume products. The lentil colour ranges from light tan to dark brown, and the latter is often referred as being a lower quality product. Furthermore, a lighter colour can be linked to a loss of nutrients or other secondary metabolites, such as polyphenolic compounds [2]. Our study discovered that the changes in lentil colour can be found during the fermentation process. It was reported that the anthocyanins can be hydrolysed by bacterial *β*-glucosidase enzymes and converted to small phenolic compounds [43]. Likewise, other flavonoid pigments (flavonols, flavones isoflavones, etc.) may be degraded by bacterial enzymes [43]. Excreted bacterial enzymes break glycosidic bonds to form aglycones and, then, the latter, after prolonged fermentation, may be further be converted into smaller phenolic compounds [43,44]. Moreover, yellow to orange pigments—carotenoids [45]—can degrade under LAB fermentation conditions, producing volatile low-molecular weight carotenoid derivatives, such as β-ionone and β-damascenone [46]. Finally, further studies are needed to explain, in detail, the colour changes in lentils during fermentation but we found correlations between the lentil colour characteristics and the main fermentation parameters (pH and LAB viable counts)—exposing that the changes occurring during lentil fermentation are very complex and should be taken into consideration during its processing.

### 3.2. Free Amino Acid Profile and γ-Aminobutyric Acid (GABA) Concentration in Lentil Wholemeal Samples

**Figure 2:**
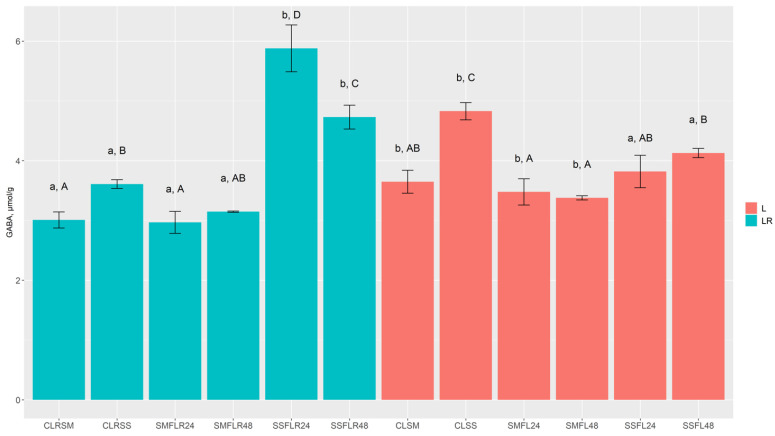
Gamma-aminobutyric acid (GABA) concentrations (µmol/g) in non-treated and fermented lentil wholemeal samples. C—control samples (non-fermented); L—lentils obtained from pure stands; LR—lentils obtained from relay incorporated with winter rye; SM—submerged conditions (lentils/water ratio 1:5, *w*/*w*); SS—submerged conditions (lentils/water ratio 1:1, *w*/*w*); SMF—fermented under submerged conditions; SSF—fermented under solid-state conditions; 24, 48—duration of fermentation (in hours). ^a,b^ Mean values denoted with different letters indicate significantly different values between the different lentil sample groups (LR and L) with the same treatment (SM, SF, SMF24, SMF48, SSF24, and SSF48); ^A–D^ mean values denoted with different letters indicate significantly different values between different treatments (SM, SF, SMF24, SMF48, SSF24, and SSF48) within the same group (LR or L). Results are statistically significant when *p* ≤ 0.05.

The non-essential free amino acid concentrations in lentil samples are presented in Table 2, and GABA concentrations in lentil samples are presented in Figure 2. The fermentation process proved to be a significant factor of reduction in arginine, asparagine, glutamine, and aspartic acid concentrations in most of the cases, while glutamic acid, glycine, alanine, and proline concentrations in fermented lentil samples were significantly higher in most cases, in comparison to respective control samples. Moreover, serine concentration in SMF samples (SMF_LR_24, SMF_LR_48, SMF_L_24, and SMF_L_48) was significantly lower, while its concentration in SSF samples (SSF_LR_24, SSF_LR_48, SSF_L_24, and SSF_L_48) was significantly higher than the respective control sample groups. Additionally, SMF_L_24, SMF_L_48, SSF_L_24, and SSF_L_48 samples contained significantly lower concentration of GABA, while its concentration was significantly higher in most of SMF and SSF LR samples, in comparison with the respective control samples.

L control samples (C_L_SM and C_L_SS) contained significantly higher concentrations of arginine, asparagine, and alanine than the LR control samples. Meanwhile, LR control samples (C_LR_SM and C_LR_SS) contained significantly higher concentrations of glutamic acid, in comparison with L control samples. Arginine concentration in almost all fermented samples was reduced to undetectable levels. L SMF samples (SMF_L_24 and SMF_L_48) contained significantly higher concentrations of glutamine, asparagine, serine, aspartic acid, glycine, alanine, proline, and GABA, in comparison to LR SMF samples (SSF_LR_24 and SSF_LR_48), while glutamic acid was significantly higher only in SMF_L_48 samples. Moreover, L SSF samples (SSF_L_24 and SSF_L_48), in comparison with SSF_LR_24 and SSF_LR_48, contained significantly higher concentrations of asparagine and aspartic acid, while glutamine and proline showed significantly higher concentrations only in SSF_L_48 samples. Furthermore, significantly higher concentrations of GABA were observed in LR SSF samples (SSF_LR_24 and SSF_LR_48), in comparison with SSF_L_24 and SSF_L_48, while only SSF_LR_24 samples contained significantly higher concentrations of glutamine, alanine, and tyrosine.

Correlations between FAA and GABA concentrations and pH and LAB count are presented in supplementary file: Table S4. Only glutamine and arginine showed positive correlations with samples’ pH (r = 0.784, *p* < 0.001 and r = 0.479, *p* = 0.003, respectively) and negative correlations with LAB count (r = −0.892, *p* < 0.001 and r = −0.593, *p* < 0.001, respectively). On the other hand, glutamic acid, aspartic acid, glycine, alanine, proline, and tyrosine showed negative correlations with lentil samples’ pH (r = −0.828, *p* < 0.001; r = −0.758, *p* < 0.001; r = −0.431, *p* = 0.009; r = −0.360, *p* = 0.031; r = −0.608, *p* < 0.001; and r = −0.442, *p* = 0.007, respectively) and negative correlations with LAB count (r = 0.869, *p* < 0.001; r = 0.794, *p* < 0.001; r = 0.369, *p* = 0.027; r = 0.329, *p* = 0.050; r = 0.472, *p* = 0.004; and r = 0.433, *p* = 0.008, respectively).

The significance of analysed factors and their interactions on FAA and GABA concentrations are presented in supplementary file: Table S3. The growing conditions factor was statistically significant for almost all nonessential FAA concentrations, except for glycine and alanine. The type of fermentation factor was statistically significant for all nonessential FAA concentrations. The fermentation duration factor was significant for almost all FAA concentrations, except for asparagine. The interaction of growing conditions * type of fermentation was significant for almost all nonessential FAAs, except for glutamic acid, aspartic acid, proline, and tyrosine. The interaction of growing conditions * fermentation duration was statistically significant for arginine, glutamine, serine, GABA, and tyrosine, while the interaction of type of fermentation * fermentation duration was significant for almost all nonessential FAAs, except for asparagine, alanine, and proline. Moreover, the interaction of growing conditions * type of fermentation * fermentation duration was statistically significant for arginine, glutamine, GABA, and tyrosine.

The essential free amino acid concentrations in lentils are depicted in Table 3. All of the fermented samples contained significantly higher concentrations of threonine, valine, phenylalanine, and leucine/isoleucine, while methionine and lysine were significantly higher for most of the fermented sample groups, in comparison to the respective control groups. However, histidine concentrations were not significantly higher in fermented sample groups, in comparison to respective control groups.

L SMF samples (SMF_L_24 and SMF_L_48) contained significantly higher concentrations of threonine, valine, and lysine, in comparison to SMF_LR_24 and SMF_LR_48, respectively, while methionine, phenylalanine, and leucine/isoleucine contained significantly higher concentrations only in SMF_L_48 samples. Furthermore, L SSF samples (SSF_L_24 and SSF_L_48), in comparison with SSF_LR_24 and SSF_LR_48, respectively, contained significantly higher concentrations of methionine, while valine, phenylalanine, leucine/isoleucine, and lysine concentrations were significantly higher only in SSF_L_48 samples. Moreover, histidine concentration in SSF_LR_24 samples was significantly higher than in the SSF_L_24 samples.

Threonine, valine, phenylalanine, leucine/isoleucine, and lysine showed negative correlations with pH (r = −0.670, *p* < 0.001; r = −0.709, *p* < 0.001; r = −0.740, *p* < 0.001; r = −0.808, *p* < 0.001; and r = −0.661, *p* < 0.001, respectively) and positive correlations with LAB count (r = 0.609, *p* < 0.001; r = 0.693, *p* < 0.001; r = 0.714, *p* < 0.001; r = 0.783, *p* < 0.001; and r = 0.537, *p* < 0.001, respectively).

Growing conditions, the type of fermentation, and fermentation duration factors were statistically significant for all essential FAAs, except histidine. The interaction of growing conditions * type of fermentation was significant for threonine, leucine/isoleucine, lysine, and histidine, while the interaction of growing conditions * fermentation duration was statistically significant for all essential FAAs, except for threonine and methionine. Moreover, the interaction of type of fermentation * fermentation duration was significant for all essential FAAs, except for lysine.

Microbial protease enzymes can effectively break down protein to produce polypeptides and free amino acids [22]. Moreover, bacteria can produce essential and non-essential amino acids by utilizing various products from the glycolysis pathway [47]. Therefore, most the changes in free amino acid composition after fermentation can be linked to the activity of bacterial proteases and other metabolic processes. The different proteolytic activities of LAB are the primary cause of the variation in the degree of protein hydrolysis [22]. Varied proteolytic activities and the ability to produce extracellular amino acids was reported for *Pediococcus acidilactici* and *Pediococcus pentosaceus* [48].

Furthermore, it has been noted that SSF, in opposite to SMF, is often associated with higher yields of fermentation products and, despite the exact reasoning not being well understood, in the literature, it is often associated with SSF conditions being similar to the natural conditions [49,50,51]. 

Arginine consumption during fermentation can be explained by BA production [52]. Asparagine and glutamine reduction could be explained by the activity of bacterial asparaginases and glutaminases in converting to aspartic and glutamic acid, respectively [53]. Glutamic acid is regarded as an intermediary amino acid that is often involved as an amino group donor for aminotransferases, while it is also consumed by the bacteria to produce GABA via decarboxylation [54]. Some species of LAB, e.g., *Lactobacillus* and *Leuconostoc*, with glutamate decarboxylase activity, also contribute to GABA production [55]. GABA is an important compound towards the inhibition of neurotransmitters, and, when implemented into the diet, can reduce sleeplessness, depression, and anxiety, enhance immunity, regulate blood pressure, etc. [54,55].

### 3.3. The Concentration of Biogenic Amines in Lentil Wholemeal Samples

Biogenic amine concentration in lentil samples is tabulated in Table 4. Tryptamine (TRIP), cadaverine (CAD), histamine (HIS), and tyramine (TYR) were not detected in any lentil groups. Phenylethylamine (PHE) was found only in SMF_LR_24 samples. In most of the cases, fermented samples contained higher concentrations of other detected biogenic amines—chiefly, putrescine (PUT), spermidine (SPRMD), and spermine (SPRM)—in comparison to the respective sample control groups. In this regard, total BA concentration in L and LR SMF and SSF sample groups were significantly higher in than the respective control sample groups.

All factors and their interactions were statistically significant on PHE concentration in lentil samples (see supplementary file: Table S6). Moreover, all factors and the growing conditions * type of fermentation interaction were statistically significant for PUT concentration. Almost all factors and the interactions thereof, except type of fermentation, were statistically significant for SPRMD concentration. The type of fermentation, the growing conditions * type of fermentation interaction, and the growing conditions * fermentation duration interaction were statistically significant for SPRM concentration in lentil samples. The correlation analysis (see supplementary file: Table S5) discovered that only PUT and SPRM possessed strong and weak positive correlations with LAB viable counts (r = 0.761, *p* < 0.001 and r = 0.331, *p* = 0.049, respectively), while only PUT showed a significant strong positive correlation with the pH of the lentils (r = 0.719, *p* < 0.001). Strong and weak negative correlations of PUT with arginine and glutamine (r = −0.686, *p* < 0.001 and r = −0.341, *p* = 0.042, respectively) were established. Moreover, strong positive correlations of PUT with glutamic and aspartic acids, valine, phenylalanine, leucine/isoleucine, and lysine (r = 0.734, *p* < 0.001; r = 0.881, *p* < 0.001; r = 0.614, *p* < 0.001; r = 0.639, *p* < 0.001; r = 0.697, *p* < 0.001; and r = 0.583, *p* < 0.001, respectively), and moderate positive correlations with threonine, glycine, and proline (r = 0.594, *p* < 0.001; r = 0.349, *p* = 0.037; and r = 0.468, *p* = 0.004, respectively) were found. SPRMD showed moderate positive correlations with GABA and tyrosine (r = 0.424, *p* = 0.010; and r = 0.534, *p* < 0.001, respectively). Also, a weak positive correlation of SPRMD with methionine (r = 0.356, *p* = 0.033) was observed. Strong positive correlations were found between SPRM and alanine, proline, glycine, phenylalanine, and histidine (r = 0.787, *p* < 0.001; r = 0.604, *p* < 0.001; r = 0.644, *p* < 0.001; r = 0.610, *p* < 0.001; and r = 0.668, *p* < 0.001, respectively). Furthermore, moderate positive correlations between SPRM and glutamic acid, serine, threonine, GABA, leucine/isoleucine, tyrosine, valine, and lysine (r = 0.461, *p* = 0.005; r = 0.430, *p* = 0.009; r = 0.563, *p* < 0.001; r = 0.566, *p* < 0.001; r = 0.425, *p* = 0.010; r = 0.466, *p* = 0.004; r = 0.520, *p* = 0.001; and r = 0.575, *p* < 0.001, respectively) were established. Yet, a weak positive correlation between SPRM and glutamine (r = 0.354, *p* = 0.034) was observed.

Aromatic and heterocyclic biogenic amines (TRIP, PHE, HIS, and TYR) are produced from the respective amino acids (i.e., tryptophan, phenylalanine, histidine, and tyrosine, respectively) by decarboxylation with enzymes that are produced by bacteria [52]. Certain LAB, e.g., *Lactococci, Pediococcus*, and *Lactobacilli*, which are decarboxylase-positive, may result in biogenic amine synthesis during fermentation processes [56]. Some strains of *Pediococcus* spp. have been found to accumulate cadaverine and tyramine in alcoholic beverages at low levels [52]. The aliphatic amine PUT can be formed from arginine via multiple pathways, e.g., arginine decarboxylation to agmatine, which is converted to PUT via agmatine deaminase and PUT carbamoyl transferase; arginine deaminization to citrulline and conversion to PUT via ornithine carbamoyl transferase; arginine conversion to ornithine via arginase/arginine ureohydrolase and conversion to PUT via ornithine decarboxylase; and decarboxylation to agmatine and conversion to PUT via agmatinase/agmatine ureohydrolase. CAD is formed from lysine via lysine decarboxylase [57]. SPRMD is formed from PUT via spermidine synthase and SPRM is formed from SPRMD via spermine synthase [58]. The Food and Drug Administration (FDA) of the United Sates of America (USA) and the Food and Agriculture Organization (FAO) of the United Nations (UN), with the World Health Organization (WHO), proposed a maximum acceptable histamine limit of 50 mg/kg in fish and food products [59]. On the other hand, according to a rat model, the no observed adverse effect level (NOAEL) was set at 2000 ppm for tyramine, putrescine, and cadaverine, 1000 ppm for spermidine, and 200 ppm for spermine [60].

### 3.4. Fatty Acid Composition in Lentil Wholemeal Samples

The results of fatty acid profiles in lentil samples are presented in Table 5. Linoleic acid (C18:2) was the most abundant fatty acid in all of the lentil samples, ranging, on average, from 41.3 to 50.3%. The least abundant fatty acid detected in all samples was saturated stearic acid (C18:0), ranging, on average, from 1.63 to 3.29%.

Palmitic acid’s (C16:0) relative concentration in the SMF and SSF LR groups (SMF_LR_24, SMF_LR_48, SSF_LR_24, and SSF_LR_48) was not significantly different from the respective control groups, while SMF_L_24, SSF_L_24, and SSF_L_48 samples contained significantly higher percentages of C16:0, in comparison with respective control. Relative concentrations of C18:0 in SMF and SSF LR samples were not significantly different from the respective control groups (except in SMF_LR_48 samples). Moreover, C18:0 relative concentrations in SMF and SSF L samples were significantly higher only in the SMF_LR_48, SMF_L_24, and SSF_L_48 samples, in comparison to respective control groups. Only the SMF_LR_24 samples contained significantly lower relative percentages of 9-octadecenoic acid (C18:1), in comparison with the respective control group, while other fermented samples did not show significant differences. Similarly, the C18:1 concentration in fermented L samples did not show significant differences when compared to the respective control groups. Furthermore, only SMF_L_24 samples contained a significantly different relative concentration of C18:2, in comparison to the respective controls. α-linolenic acid’s (C18:3 α) relative concentration in SMF and SSF LR samples was not different from the respective controls. On the other hand, its relative concentrations in SMF_L_24, SMF_LR_24, SSF_L_24, and SSF_L_48 sample groups were lower than those in the respective controls. Tetradecanoic acid (C14:0), eicosanoic acid (C20:0), and *cis*-11-eicosenoic acid (C20:1) were only detected in fermented sample groups. The factor of growing conditions was statistically significant in almost all the content of fatty acids in lentils but C16:0 and C18:0 (see supplementary file: Table S8). The type of fermentation was a statistically significant factor for almost all fatty acids content except C18:0 and C18:2. Fermentation duration was a statistically significant factor for most fatty acids content except C18:0, C18:1, and C18:2. The factor interactions (growing conditions * type of fermentation and type of fermentation * fermentation duration) were shown to be statistically significant for the most of the fatty acids, but not for C18:1 and C18:2, while the growing conditions * fermentation duration factor’s interaction was only not statistically significant for C18:1 content in lentils. Furthermore, the interaction of the factors growing conditions * type of fermentation * fermentation duration was statistically significant for all fatty acid content in lentils. A weak positive correlation was found between C18:0 concentration and LAB viable counts (r = 0.363, *p* = 0.030) in lentils (see supplementary file: Table S7).

Saturated (SFA), monounsaturated (MUFA), and polyunsaturated (PUFA) fatty acids and omega-3, omega-6, and omega-9 fatty acid concentrations (% of the total fat content) of lentils are displayed in Table 6. As expected, the predominant groups of fatty acids in lentils were PUFA and omega-6. Only L fermented samples often showed significantly higher SFA contents than in the respective control groups. Other fatty acid groups were not significantly different, in comparison to the non-treated and fermented lentil groups.

It has been reported that C16:0, C18:0, C18:1, C18:2, and C18:3 α are the main fatty acids detected in lentils (*Lens culinaris*). The major fatty acid detected in lentil samples was C18:2, as noted in the literature, and, furthermore, driving PUFA were described to be most abundant group of fatty acids in lentils [5,61,62]. Most of the established differences observed between the L and LR sample groups (fermented and non-fermented) can be explained by differences in type of cultivar, genotype, or growing conditions [63,64].

### 3.5. Volatile Compound Profile in Lentil Wholemeal Samples

**Figure 3:**
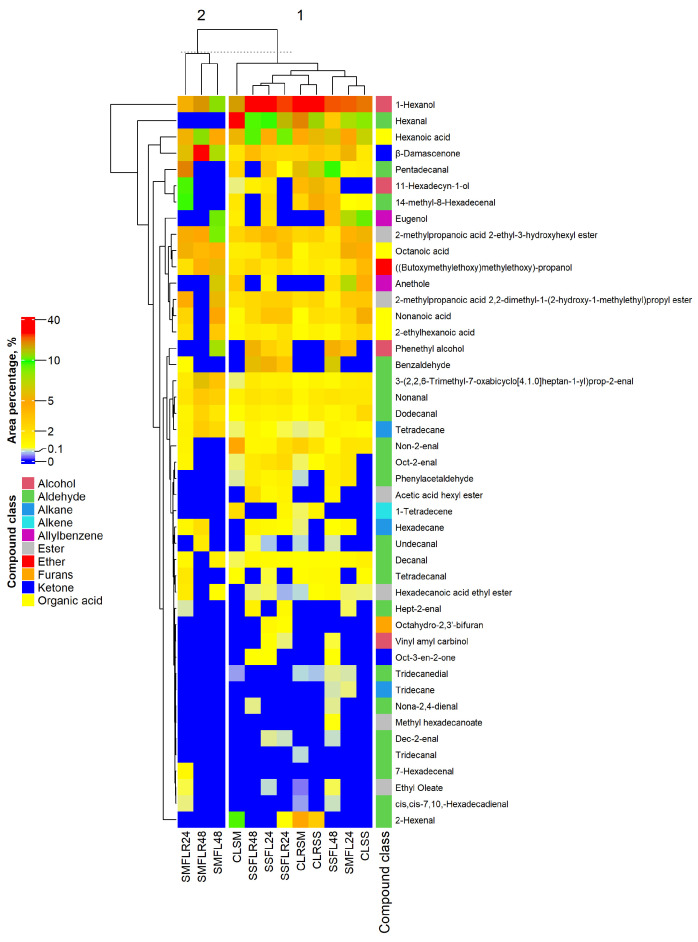
Volatile compound (VC) profile (% of the total volatile compounds content) in lentils. C—control samples (non-fermented); L—lentils obtained from pure stands; LR—lentils obtained from relay incorporated with winter rye; SM—submerged conditions (lentils/water ratio 1:5, *w*/*w*); SS—submerged conditions (lentils/water ratio 1:1, *w*/*w*); SMF—fermented under submerged conditions; SSF—fermented under solid state conditions; 24, 48—duration of fermentation (in hours).

The volatile compound profiles (% of the total identified VC content) are presented in Figure 3 (see supplementary file: Table S11). The most abundant VCs (the total area percentage of which was at least 10% in one of the sample groups) were 1-hexanol, hexanal, hexanoic acid, β-damascenone, pentadecanal, 11-hexadecyn-1-ol, 14-methyl-8-hexadecenal, and eugenol. The most abundant VCs presented as various classes of chemical compounds: alcohols (1-hexanol and 11-hexadecyn-1-ol), aldehydes (hexanal, pentadecanal, and 14-methyl-8-hexadecenal), organic acids (hexanoic acid), ketones (β-damascenone), and allylbenzenes (eugenol). Most of the identified VCs with lower abundances (i.e., an area percentage under 10%) in lentils are aldehydes. Furthermore, in almost all cases, identified VCs with lower abundances in the sample control groups had significantly higher abundances in fermented sample groups. Moreover, the complexity of VC composition in the SSF fermented sample groups (for example, there were 30, 35, 28, and 39 VCs detected in the SSF_LR_24, SSF_L_24, SSF_LR_48, and SSF_L_48 samples, respectively) was higher than in the respective control groups (i.e., 25 and 22 VCs were detected in the C_LR_SS and C_L_SS samples, respectively). In addition, the SSF_LR_24 and SSF_LR_48 sample groups contained nine newly formed VCs, while the SSF_L_24 and SSF_L_48 samples contained thirteen and seventeen newly formed VCs, respectively. Moreover, between 38.5 and 55.6% of newly formed VCs detected in SSF samples were aldehydes. However, in SMF sample groups, only three, five, and two newly formed VCs were detected in the SMF_LR_24, SMF_L_24, and SMF_L_48 sample groups, while, in the SMF_LR_48 sample group, the VC profile was similar to the non-fermented samples. Most of the SMF samples contained VCs belonging to aldehydes and alkanes for the SMF_LR_24 and SMF_L_24 sample groups, while alcohols and ketones were the largest newly formed VC classes in the SMF_L_48 sample group.

Most of VCs detected in lentils are known to be associated with specific flavour and aroma characteristics. The 1-hexanol is described having light apple, sweet, fruity, ethereal, and herbal flavour notes with herbal and oily aroma characteristics [65,66]. Some of the most abundant aldehydes—hexanal and pentadecanal—have similar flavour and aroma traits and are described as fatty, grassy, fresh, and green [65,66,67]. Organic acids, such as hexanoic acid, are described as having sour, fatty, and cheesy flavours and aromas [65]. β-Damascenone contributes to woody, fruity, stewed apple, and sweet aroma and flavour notes [65]. Eugenol is known to contribute to flavour and aroma with clove, spicy, and honeylike notes [65].

The aliphatic aldehydes and ketones, such as hexanal or oct-3-en-2-one, are associated with fatty acid oxidation [68]. The detected aromatic aldehydes and alcohols, benzaldehyde phenylacetaldehyde and phenylethyl alcohol, showed a statistically significant correlation with the aromatic amino acid phenylalanine (see supplementary file: Table S11). Aromatic aldehydes and alcohols are associated with the catabolism of aromatic amino acids; in this case, phenylalanine [69]. The respective amino acid is converted to α-keto acid by the respective aminotransferase, which can be converted to aldehyde by decarboxylases [53,69]. Furthermore, the respective aldehyde can be oxidised to form carboxylic acids or reduced to form alcohols [53,68]. The ester formation has four main biosynthesis pathways, as follows: alcohol and acid conversion to ester via esterase; hemiacetal, which is formed via spontaneous reaction between aldehyde and alcohol, with dehydrogenation via its dehydrogenases; ketone conversion to ester via Baeyer–Villiger monooxygenases; and alcohol and acyl-CoA conversion to ester via alcohol acyltransferases [70]. The production of hydrocarbons, such as tetradecane, tridecane, or 1-tetradecene, is associated with fatty acid catabolism [71]. Finally, all the factors and their interactions were statistically significant for most of the VCs in lentils (see supplementary file: Table S12).

### 3.6. Micro- and Macro-Elements Concentration in Lentil Wholemeal Samples

The concentrations of micro- and macro-elements in lentil samples are presented in Table 7. Almost all concentrations of macro-elements were significantly higher in L samples, in comparison with LR ones, except magnesium. Moreover, of all essential micro-elements, only phosphorus, manganese, and selenium concentrations in the L sample group were significantly higher than in the LR group. Moreover, iron and nickel concentrations in the LR samples were significantly higher than in the L samples.

Lentils are defined as a leguminous crop, containing significant amounts of macro-elements and essential micro-elements, such as phosphorus, potassium, iron, and zinc [72,73]. Furthermore, the concentration of essential microelements, such as iron, is high enough for the addition of lentils in a diet to prevent iron deficiency anaemia [5]. The other minerals that are plentiful in lentils are crucial for important biological functions in humans [74]. These functions include, but are not limited to, the following: muscle and nerve functioning (Mg and Ca); cell growth, function, and energy production (P); electrolyte balance and muscle contraction (Na); the metabolism of fat and carbohydrates (Cr); the structural components of many enzymes (Mn, Zn, and Cu); etc. [74,75].

According to various sources in the literature, the concentrations of various essential microelements can vary within the ranges of 2000–5300 mg/kg, 0.12–0.60 mg/kg, 10.5–29.0 mg/kg, 49–81.4 mg/kg, 1.1–1.8 mg/kg, 9.4–14.3 mg/kg, 36.7–64.2 mg/kg, and 0.18–1.6 mg/kg for phosphorus, chromium, manganese, iron, nickel, copper, zinc, and selenium, respectively [1,72,76,77,78]. The mineral composition of lentils can be affected by differences in growing conditions (e.g., phosphorus and other mineral fertilization, water availability, and environmental conditions), plant origin (e.g., genotype and cultivar), or other parameters (e.g., harvesting time) [73,79,80,81,82,83].

However, the aforementioned micronutrients have their bioavailability restrained due to the existing antinutrient factors (phytates, oxalates, and phenolic compounds) [72,84]. Mineral bioavailability can be enhanced by employing certain strategies, such as genetic manipulation, dehulling, soaking, enzymatic treatment, or fermentation [5,72,84,85]. The bioavailability enhancement of minerals via fermentation can be explained by the disruption of plant cell wall structures, production of phytase enzymes, or even the activation of endogenous phytase enzymes, due to the existing low pH environment [26,86,87].

## 4. Conclusions

This study showed that the *Pediococcus acidilactici* strain was suitable for the fermentation of lentils, once the LAB count in all fermented lentil samples was higher than 7.00 log_10_ CFU/g and a strong negative correlation between pH and LAB viable counts in lentils (r = −0.886, *p* < 0.001) was established. The analysed factors (growing conditions, type of fermentation, and fermentation duration) were statistically significant in most of the lentils’ chromaticity parameters. Solid-state fermented (SSF) lentils showed a more complex volatile compound profile (between nine and seventeen new VCs were formed), whereas, in submerged fermented samples (SMF), between two and five newly formed VCs were detected. Comparing lentils grown in pure stands (L) and lentils grown in relay intercropped with winter rye (LR), L contained significantly higher concentrations of Na, K, and Ca and the essential microelements P, Mn, and Se, while LR contained significantly higher concentrations of the essential microelements Fe and Ni. The most of free amino acid concentrations, as well as the total biogenic amine content, increased in lentils after SMF and SSF. The most abundant fatty acid in lentils was C18:2 (which ranged from 41.3 to 50.3% of total FA content). The SSF LR samples contained the most GABA, while all the analysed factors and their interactions were statistically significant for GABA concentration in lentils. This research effort showed that fermentation is a useful biotechnology for increasing the biological value of lentils; however, biogenic amine concentration should be taken into consideration. Overall, this study will contribute to the creation of a database on the micro- and macro-elements composition of new varieties of lentils, grown either in pure stands or relay intercropped with winter rye.

## Figures and Tables

**Table 1:** Acidity, microbiological, and chromaticity characteristics in lentil wholemeal samples.

Lentil Samples	Sample Groups	pH	LAB Viable Counts, log_10_ CFU/g	Colour Coordinates, NBS		
				L*	a*	b*
LR	C_LR_SM	6.12 ± 0.03 ^b,E^	4.80 ± 0.23 ^b,A^	68.0 ± 0.31 ^a,E^	1.85 ± 0.11 ^b,B^	31.0 ± 0.29 ^a,D^
	C_LR_SS	6.03 ± 0.01 ^b,D^	4.60 ± 0.19 ^a,A^	67.9 ± 0.22 ^a,E^	1.78 ± 0.12 ^b,B^	31.3 ± 0.23 ^a,D^
	SMF_LR_24	5.20 ± 0.03 ^a,C^	7.98 ± 0.21 ^b,BC^	63.2 ± 0.27 ^a,C^	1.08 ± 0.07 ^a,A^	23.5 ± 0.41 ^a,C^
	SMF_LR_48	4.36 ± 0.02 ^a,A^	8.27 ± 0.22 ^a,C^	61.6 ± 0.43 ^b,B^	2.96 ± 0.14 ^a,C^	21.6 ± 0.36 ^b,B^
	SSF_LR_24	5.22 ± 0.03 ^b,C^	7.63 ± 0.23 ^a,B^	64.0 ± 0.25 ^a,D^	1.19 ± 0.08 ^a,A^	24.3 ± 0.25 ^a,C^
	SSF_LR_48	4.72 ± 0.02 ^b,B^	8.14 ± 0.19 ^a,BC^	50.9 ± 0.19 ^a,A^	5.34 ± 0.21 ^a,D^	15.6 ± 0.21 ^a,A^
L	C_L_SM	6.02 ± 0.01 ^a,E^	4.42 ± 0.15 ^a,A^	69.6 ± 0.25 ^b,E^	1.01 ± 0.06 ^a,A^	34.7 ± 0.37 ^b,E^
	C_L_SS	5.94 ± 0.03 ^a,D^	4.87 ± 0.24 ^a,A^	69.4 ± 0.39 ^b,E^	1.08 ± 0.05 ^a,A^	34.5 ± 0.41 ^b,E^
	SMF_L_24	5.58 ± 0.02 ^b,C^	7.61 ± 0.20 ^a,BC^	67.7 ± 0.26 ^b,D^	1.98 ± 0.12 ^b,B^	32.3 ± 0.32 ^b,D^
	SMF_L_48	4.45 ± 0.02 ^b,A^	8.41 ± 0.19 ^a,D^	58.8 ± 0.32 ^a,B^	5.12 ± 0.23 ^b,C^	18.6 ± 0.09 ^a,B^
	SSF_L_24	4.71 ± 0.03 ^a,B^	7.30 ± 0.22 ^a,B^	66.8 ± 0.42 ^b,C^	1.68 ± 0.14 ^b,B^	29.7 ± 0.14 ^b,C^
	SSF_L_48	4.46 ± 0.01 ^a,B^	8.10 ± 0.18 ^a,CD^	54.5 ± 0.29 ^b,A^	7.03 ± 0.25 ^b,D^	17.6 ± 0.11 ^b,A^

C—control samples (non-fermented); L—lentils obtained from pure stands; LR—lentils obtained from relay incorporated with winter rye; SM—submerged conditions (lentils/water ratio 1:5, *w*/*w*); SS—submerged conditions (lentils/water ratio 1:1, *w*/*w*); SMF—fermented under submerged conditions; SSF—fermented under solid-state conditions; 24, 48—duration of fermentation (in hours). L*—brightness or (-) darkness; a*—redness or (-) greenness; b*—yellowness or (-) blueness; NBS—National Bureau of Standards units; LAB—lactic acid bacteria; CFU—colony-forming units. Data are represented as means (n = 3) ± standard errors (SE). ^a,b^ Mean values denoted with different letters indicate significantly different values between the different lentil sample groups (LR and L) with the same treatment (SM, SF, SMF24, SMF48, SSF24, and SSF48); ^A–E^ mean values denoted with different letters indicate significantly different values between different treatments (SM, SF, SMF24, SMF48, SSF24, and SSF48) within the same group (LR or L). Results are statistically significant when *p* ≤ 0.05.

**Table 2:** Concentration of non-essential free amino acids (FAAs) in lentil wholemeal samples.

Lentil Samples	Arginine	Glutamine	Asparagine	Glutamic Acid	Serine	Aspartic Acid	Glycine	Alanine	Proline	Tyrosine
	µmol/g									
LR										
C_LR_SM	1.86 ± 0.028 ^a,B^	0.943 ± 0.042 ^a,B^	1.60 ± 0.019 ^a,BC^	3.42 ± 0.085 ^b,A^	0.819 ± 0.057 ^a,B^	nd	1.50 ± 0.091 ^a,A^	3.62 ± 0.069 ^a,A^	0.750 ± 0.027 ^a,A^	0.476 ± 0.039 ^a,A^
C_LR_SS	2.28 ± 0.11 ^a,C^	1.96 ± 0.086 ^a,E^	1.84 ± 0.085 ^a,CD^	4.16 ± 0.134 ^b,B^	1.03 ± 0.012 ^a,B^	0.738 ± 0.027 ^a,B^	1.91 ± 0.023 ^a,C^	6.69 ± 0.176 ^a,C^	0.990 ± 0.043 ^a,B^	0.679 ± 0.009 ^b,BC^
SMF_LR_24	nd	0.172 ± 0.014 ^a,A^	1.17 ± 0.026 ^a,A^	4.85 ± 0.073 ^a,C^	0.128 ± 0.017 ^a,A^	0.879 ± 0.052 ^a,C^	1.58 ± 0.027 ^a,AB^	5.09 ± 0.078 ^a,B^	0.760 ± 0.053 ^a,A^	0.635 ± 0.011 ^b,B^
SMF_LR_48	nd	0.178 ± 0.01 ^a,A^	1.33 ± 0.023 ^a,AB^	5.00 ± 0.165 ^a,C^	nd	1.08 ± 0.076 ^a,D^	1.73 ± 0.026 ^a,B^	5.23 ± 0.077 ^a,B^	0.960 ± 0.042 ^a,B^	0.627 ± 0.013 ^a,B^
SSF_LR_24	0.75 ± 0.041 ^a,A^	1.70 ± 0.059 ^b,D^	1.97 ± 0.175 ^a,D^	4.73 ± 0.120 ^a,C^	1.44 ± 0.090 ^a,C^	0.439 ± 0.012 ^a,A^	2.26 ± 0.057 ^a,D^	8.43 ± 0.652 ^b,D^	1.37 ± 0.092 ^a,C^	0.613 ± 0.011 ^b,B^
SSF_LR_48	nd	1.11 ± 0.039 ^a,C^	1.90 ± 0.138 ^a,D^	6.02 ± 0.074 ^a,D^	1.67 ± 0.171 ^a,D^	0.949 ± 0.036 ^a,C^	2.67 ± 0.108 ^a,E^	9.01 ± 0.755 ^a,D^	1.56 ± 0.032 ^a,D^	0.753 ± 0.053 ^a,C^
L										
C_L_SM	2.99 ± 0.152 ^b,A^	1.35 ± 0.027 ^b,B^	3.63 ± 0.043 ^b,B^	2.14 ± 0.051 ^a,A^	0.893 ± 0.077 ^a,B^	nd	1.62 ± 0.093 ^a,A^	3.90 ± 0.09 ^b,A^	0.752 ± 0.072 ^a,A^	0.501 ± 0.042 ^a,A^
C_L_SS	4.35 ± 0.31 ^b,B^	2.00 ± 0.112 ^a,C^	4.68 ± 0.300 ^b,C^	2.73 ± 0.029 ^a,A^	1.25 ± 0.089 ^b,C^	nd	2.21 ± 0.080 ^b,BC^	8.32 ± 0.09 ^b,D^	1.11 ± 0.020 ^b,B^	0.634 ± 0.014 ^a,B^
SMF_L_24	nd	0.694 ± 0.007 ^b,A^	3.14 ± 0.201 ^b,A^	4.95 ± 0.163 ^a,B^	0.269 ± 0.003 ^b,A^	1.39 ± 0.016 ^b,B^	1.88 ± 0.036 ^b,AB^	5.90 ± 0.393 ^b,B^	0.890 ± 0.065 ^b,A^	0.595 ± 0.009 ^a,B^
SMF_L_48	nd	0.708 ± 0.051 ^b,A^	3.23 ± 0.038 ^b,AB^	5.67 ± 0.311 ^b,BC^	0.427 ± 0.021 ^a,A^	1.57 ± 0.103 ^b,C^	2.01 ± 0.117 ^b,B^	6.29 ± 0.162 ^b,BC^	1.15 ± 0.053 ^b,B^	0.632 ± 0.031 ^a,B^
SSF_L_24	nd	1.20 ± 0.053 ^a,B^	3.41 ± 0.237 ^b,AB^	5.10 ± 0.411 ^a,B^	1.34 ± 0.094 ^a,C^	0.928 ± 0.035 ^b,A^	2.05 ± 0.173 ^a,B^	7.02 ± 0.246 ^a,C^	1.44 ± 0.015 ^a,C^	0.495 ± 0.022 ^a,A^
SSF_L_48	nd	1.25 ± 0.016 ^b,B^	3.53 ± 0.049 ^b,AB^	6.15 ± 0.437 ^a,C^	1.74 ± 0.031 ^a,D^	1.60 ± 0.044 ^b,C^	2.51 ± 0.191 ^a,C^	8.23 ± 0.711 ^a,D^	1.70 ± 0.085 ^b,D^	0.818 ± 0.019 ^a,C^

C—control samples (non-fermented); L—lentils obtained from pure stands; LR—lentils obtained from relay incorporated with winter rye; SM—submerged conditions (lentils/water ratio 1:5, *w*/*w*); SS—submerged conditions (lentils/water ratio 1:1, *w*/*w*); SMF—fermented under submerged conditions; SSF—fermented under solid-state conditions; 24, 48—duration of fermentation (in hours); nd—not detected. ^a,b^ Mean values denoted with different letters indicate significantly different values between the different lentil sample groups (LR and L) with the same treatment (SM, SF, SMF24, SMF48, SSF24, and SSF48); ^A–E^ mean values denoted with different letters indicate significantly different values between different treatments (SM, SF, SMF24, SMF48, SSF24, and SSF48) within the same group (LR or L). Results are statistically significant when *p* ≤ 0.05.

**Table 3:** Concentrations of essential free amino acids (FAA) in lentil wholemeal samples.

Lentil Samples	Threonine	Valine	Methionine	Phenylalanine	Leucine/Isoleucine	Lysine	Histidine
	µmol/g						
LR							
C_LR_SM	1.15 ± 0.091 ^a,A^	1.080 ± 0.086 ^a,A^	0.115 ± 0.008 ^a,A^	0.484 ± 0.040 ^a,A^	1.27 ± 0.019 ^a,A^	1.69 ± 0.118 ^a,A^	0.62 ± 0.019 ^a,A^
C_LR_SS	1.46 ± 0.028 ^a,B^	1.5 ± 0.039 ^a,B^	0.195 ± 0.025 ^a,C^	0.632 ± 0.012 ^a,B^	1.77 ± 0.036 ^b,B^	2.04 ± 0.031 ^a,B^	0.86 ± 0.019 ^a,C^
SMF_LR_24	1.39 ± 0.099 ^a,B^	1.76 ± 0.112 ^a,B^	0.16 ± 0.002 ^a,B^	0.697 ± 0.020 ^a,B^	2.75 ± 0.173 ^a,CD^	1.80 ± 0.094 ^a,AB^	0.54 ± 0.012 ^a,A^
SMF_LR_48	1.47 ± 0.116 ^a,B^	1.65 ± 0.008 ^a,B^	0.152 ± 0.008 ^a,B^	0.685 ± 0.034 ^a,B^	2.56 ± 0.108 ^a,C^	1.98 ± 0.070 ^a,B^	0.58 ± 0.017 ^a,A^
SSF_LR_24	2.1 ± 0.061 ^a,C^	2.3 ± 0.145 ^a,C^	0.112 ± 0.002 ^a,A^	0.848 ± 0.075 ^a,C^	3.09 ± 0.244 ^a,D^	2.77 ± 0.086 ^a,C^	0.86 ± 0.067 ^b,C^
SSF_LR_48	2.43 ± 0.046 ^a,D^	2.98 ± 0.148 ^a,D^	0.222 ± 0.003 ^a,C^	0.912 ± 0.024 ^a,C^	4.39 ± 0.150 ^a,E^	2.90 ± 0.134 ^a,C^	0.73 ± 0.063 ^a,B^
L							
C_L_SM	1.21 ± 0.098 ^a,A^	1.122 ± 0.103 ^a,A^	0.168 ± 0.014 ^b,BC^	0.428 ± 0.032 ^a,A^	1.22 ± 0.101 ^a,A^	1.721 ± 0.113 ^a,A^	0.58 ± 0.022 ^a,AB^
C_L_SS	1.59 ± 0.023 ^b,B^	1.53 ± 0.030 ^a,B^	0.219 ± 0.005 ^a,DE^	0.638 ± 0.018 ^a,B^	1.61 ± 0.055 ^a,B^	2.24 ± 0.041 ^b,B^	1.01 ± 0.043 ^b,D^
SMF_L_24	1.69 ± 0.117 ^b,BC^	1.94 ± 0.046 ^b,C^	0.176 ± 0.012 ^a,BC^	0.680 ± 0.051 ^a,BC^	2.69 ± 0.037 ^a,C^	1.93 ± 0.014 ^b,A^	0.55 ± 0.036 ^a,A^
SMF_L_48	1.93 ± 0.047 ^b,CD^	2.05 ± 0.061 ^b,C^	0.199 ± 0.002 ^b,CD^	0.769 ± 0.025 ^b,CD^	3.48 ± 0.026 ^b,D^	2.73 ± 0.031 ^b,C^	0.64 ± 0.009 ^a,B^
SSF_L_24	2.14 ± 0.173 ^a,D^	2.36 ± 0.122 ^a,D^	0.141 ± 0.017 ^b,A^	0.834 ± 0.050 ^a,D^	3.38 ± 0.044 ^a,D^	2.78 ± 0.189 ^a,C^	0.73 ± 0.039 ^a,C^
SSF_L_48	2.52 ± 0.155 ^a,E^	3.33 ± 0.051 ^b,E^	0.254 ± 0.021 ^b,E^	1.093 ± 0.052 ^b,E^	5.47 ± 0.164 ^b,E^	3.38 ± 0.130 ^b,D^	0.73 ± 0.022 ^a,C^

C—control samples (non-fermented); L—lentils obtained from pure stands; LR—lentils obtained from relay incorporated with winter rye; SM—submerged conditions (lentils/water ratio 1:5, *w*/*w*); SS—submerged conditions (lentils/water ratio 1:1, *w*/*w*); SMF—fermented under submerged conditions; SSF—fermented under solid-state conditions; 24, 48—duration of fermentation (in hours). ^a,b^ Mean values denoted with different letters indicate significantly different values between the different lentil sample groups (LR and L) with the same treatment (SM, SF, SMF24, SMF48, SSF24, and SSF48); ^A–E^ mean values denoted with different letters indicate significantly different values between different treatments (SM, SF, SMF24, SMF48, SSF24, and SSF48) within the same group (LR or L). Results are statistically significant when *p* ≤ 0.05.

**Table 4:** Biogenic amine (BA) concentration in lentil wholemeal samples.

Lentil Samples		TRIP	PHE	PUT	CAD	HIS	TYR	SPRMD	SPRM	Total BA Content
		mg/kg								
LR	C_LR_SM	nd	nd	nd	nd	nd	nd	75.8 ± 5.06 ^a,A^	13.6 ± 1.1 ^a,A^	89.4 ± 6.2 ^a,A^
	C_LR_SS	nd	nd	14.6 ± 0.12 ^a,A^	nd	nd	nd	110.1 ± 8.53 ^a,C^	20.7 ± 1.6 ^a,B^	145.4 ± 10.3 ^a,B^
	SMF_LR_24	nd	4.9 ± 0.41 ^a,A^	36.2 ± 2.14 ^a,B^	nd	nd	nd	87.8 ± 6.87 ^a,B^	15.2 ± 1.2 ^a,A^	144.1 ± 10.6 ^a,B^
	SMF_LR_48	nd	nd	41.8 ± 3.12 ^a,C^	nd	nd	nd	84.7 ± 5.42 ^a,A,B^	13.8 ± 1.1 ^a,A^	140.3 ± 9.6 ^a,B^
	SSF_LR_24	nd	nd	32.2 ± 2.81 ^a,B^	nd	nd	nd	129 ± 8.23 ^b,D^	25.3 ± 1.6 ^b,C^	186.5 ± 12.6 ^a,C^
	SSF_LR_48	nd	nd	37.2 ± 2.89 ^a,BC^	nd	nd	nd	116.7 ± 7.29 ^b,CD^	22.4 ± 1.8 ^a,BC^	176.3 ± 12.0 ^a,C^
L	C_L_SM	nd	nd	nd	nd	nd	nd	87.3 ± 5.32 ^b,A^	nd	87.3 ± 5.3 ^a,A^
	C_L_SS	nd	nd	22.1 ± 1.83 ^b,A^	nd	nd	nd	117.1 ± 8.36 ^a,C^	23.9 ± 1.5 ^a,C^	163.1 ± 11.7 ^a,B^
	SMF_L_24	nd	nd	79.5 ± 5.45 ^b,B^	nd	nd	nd	96.5 ± 7.21 ^a,AB^	16.4 ± 1.3 ^a,A^	192.4 ± 14.0 ^b,BC^
	SMF_L_48	nd	nd	97.4 ± 6.84 ^b,C^	nd	nd	nd	99.6 ± 6.81 ^b,B^	17.9 ± 1.6 ^b,AB^	214.9 ± 15.3 ^b,C^
	SSF_L_24	nd	nd	71.3± 5.42 ^b,B^	nd	nd	nd	103.4 ± 8.25 ^a,C^	19.5 ± 1.7 ^a,B^	194.2 ± 1.1 ^a,BC^
	SSF_L_48	nd	nd	77.2 ± 6.12 ^b,B^	nd	nd	nd	101.5 ± 7.46 ^a,BC^	19.3 ± 1.8 ^a,AB^	198 ± 15.4 ^a,C^

C—control samples (non-fermented); L—lentil obtained from pure stands; LR—lentil obtained from relay incorporated with winter rye; SM—submerged conditions (lentils/water ratio 1:5, *w*/*w*); SS—submerged conditions (lentils/water ratio 1:1, *w*/*w*); SMF—fermented under submerged conditions; SSF—fermented under solid-state conditions; 24, 48—duration of fermentation (in hours). TRIP—tryptamine; PHE—phenylethylamine; PUT—putrescine; CAD—cadaverine; HIS—histamine; TYR—tyramine; SPRMD—spermidine; SPRM—spermine; BA—biogenic amines. nd—not detected. ^a,b^ Mean values denoted with different letters indicate significantly different values between the different lentil sample groups (LR and L) with the same treatment (SM, SF, SMF24, SMF48, SSF24, and SSF48); ^A–D^ mean values denoted with different letters indicate significantly different values between different treatments (SM, SF, SMF24, SMF48, SSF24, and SSF48) within the same group (LR or L). Results are statistically significant when *p* ≤ 0.05.

**Table 5:** Fatty acid (FA) profiles in lentil wholemeal samples.

		C14:0	C16:0	C18:0	C18:1	C18:2	C18:3 α	C20:0	C20:1
		Fatty Acids Concentration, % of Total Fat Content							
LR	C_LR_SM	nd	17.5 ± 1.1 ^a,AB^	1.9 ± 0.21 ^a,A^	18.0 ± 0.32 ^a,B^	48.1 ± 0.43 ^a,A^	14.6 ± 1.23 ^a,AB^	nd	nd
	C_LR_SS	nd	18.6 ± 1.0 ^b,B^	2.39 ± 0.13 ^b,B^	16.9 ± 1.20 ^a,AB^	47.3 ± 3.77 ^a,A^	14.8 ± 1.04 ^a,AB^	nd	nd
	SMF_LR_24	nd	15.3 ± 1.2 ^a,A^	1.9 ± 0.10 ^a,A^	15.8 ± 0.71 ^a,A^	51.2 ± 1.07 ^b,A^	15.8 ± 0.83 ^b,B^	nd	nd
	SMF_LR_48	nd	17.0 ± 0.56 ^b,A^	2.46 ± 0.14 ^b,B^	16.5 ± 0.45 ^a,AB^	48.0 ± 2.21 ^a,A^	16.0 ± 0.67 ^b,B^	nd	nd
	SSF_LR_24	nd	18.3 ± 0.29 ^a,B^	2.38 ± 0.22 ^b,B^	18.0 ± 0.51 ^a,B^	47.4 ± 1.34 a,^A^	14.0 ± 0.33 ^b,A^	nd	nd
	SSF_LR_48	nd a	18.0 ± 0.35 ^a,B^	2.56 ± 0.09 ^a,B^	17.6 ± 0.68 ^a,B^	47.4 ± 1.05 ^a,A^	14.0 ± 0.48 ^b,A^	0.33 ± 0.03 ^a,A^	nd
L	C_L_SM	nd	16.3 ± 0.29 ^a,B^	1.89 ± 0.38 ^a,AB^	19.2 ± 0.69 ^b,AB^	48.2 ± 1.10 ^a,B^	14.4 ± 0.82 ^a,B^	nd	nd
	C_L_SS	nd	16.2 ± 0.58 ^a,B^	1.63 ± 0.28 ^a,A^	19.8 ± 1.12 ^b,AB^	48.4 ± 3.82 ^a,B^	14.0 ± 0.65 ^a,B^	nd	nd
	SMF_L_24	2.63 ± 0.23 ^A^	20.3 ± 1.25 ^b,D^	3.29 ± 0.24 ^b,C^	20.4 ± 0.68 ^b,B^	41.3 ± 2.23 ^a,A^	12.1 ± 0.41 ^a,A^	nd	nd
	SMF_L_48	nd	14.1 ± 0.54 ^a,A^	1.83 ± 0.21 ^a,AB^	19.0 ± 0.63 ^b,A^	50.3 ± 2.28 ^a,B^	14.8 ± 0.43 ^a,B^	nd	nd
	SSF_L_24	nd	17.9 ± 0.42 ^a,C^	1.94 ± 0.15 ^a,AB^	20.3 ± 1.30 ^b,AB^	46.6 ± 2.40 ^a,B^	12.6 ± 0.55 ^a,A^	nd	0.671 ± 0.03 ^a,A^
	SSF_L_48	nd	17.5 ± 0.45 ^a,C^	2.31 ± 0.28 ^a,B^	21.2 ± 1.11 ^b,B^	46.4 ± 3.31 ^a,AB^	12.6 ± 0.48 ^a,A^	nd	nd

C—control samples (non-fermented); L—lentils obtained from pure stands; LR—lentils obtained from relay incorporated with winter rye; SM—submerged conditions (lentils/water ratio 1:5, *w*/*w*); SS—submerged conditions (lentils/water ratio 1:1, *w*/*w*); SMF—fermented under submerged conditions; SSF—fermented under solid state conditions; 24, 48—duration of fermentation (in hours). C14:0—tetradecanoic acid; C16:0—palmitic acid; C18:0—stearic acid; C18:1—9-octadecenoic acid; C18:2—linoleic acid; C18:3 α—α-linolenic acid; C20:0—eicosanoic acid; C20:1—*cis*-11-eicosenoic acid. nd—not detected. ^a,b^ Mean values denoted with different letters indicate significantly different values between the different lentil sample groups (LR and L) with the same treatment (SM, SF, SMF24, SMF48, SSF24, and SSF48); ^A–D^ Mean values denoted with different letters indicate significantly different values between different treatments (SM, SF, SMF24, SMF48, SSF24, and SSF48) within the same group (LR or L). Results are statistically significant when *p* ≤ 0.05.

**Table 6:** Saturated (SFA), monounsaturated (MUFA), and polyunsaturated (PUFA) fatty acids, and omega-3, omega-6, and omega-9 contents in lentil wholemeal samples.

Lentil Samples		SFA	MUFA	PUFA	Omega-3	Omega-6	Omega-9
		Concentration, % of Total Fat Content					
LR	C_LR_SM	19.4 ± 1.67 ^a,AB^	18.0 ± 1.38 ^a,A^	62.6 ± 2.88 ^a,A^	14.6 ± 0.58 ^a,A^	48.1 ± 4.48 ^a,A^	18.0 ± 1.45 ^a,B^
	C_LR_SS	20.9 ± 2.04 ^b,B^	16.9 ± 0.87 ^a,A^	62.2 ± 5.11 ^a,A^	14.8 ± 1.12 ^a,AB^	47.3 ± 3.14 ^a,A^	16.9 ± 0.783 ^a,AB^
	SMF_LR_24	17.2 ± 0.72 ^a,A^	15.8 ± 1.11 ^a,A^	67.1 ± 2.21 ^b,A^	15.8 ± 0.84 ^b,AB^	51.2 ± 3.10 ^b,A^	15.8 ± 0.67 ^a,A^
	SMF_LR_48	19.5 ± 0.76 ^b,B^	16.5 ± 0.71 ^a,A^	64.0 ± 3.08 ^a,A^	16.0 ± 0.50 ^b,B^	48.0 ± 1.54 ^a,A^	16.5 ± 0.89 ^a,AB^
	SSF_LR_24	20.7 ± 1.26 ^a,B^	18.0 ± 1.64 ^a,A^	61.3 ± 5.72 ^a,A^	14.0 ± 1.20 ^a,A^	47.4 ± 3.65 ^a,A^	18.0 ± 0.88 ^a,B^
	SSF_LR_48	20.9 ± 0.75 ^a,B^	17.6 ± 1.26 ^a,A^	61.4 ± 5.42 ^a,A^	14.0 ± 1.06 ^a,A^	47.4 ± 2.14 ^a,A^	17.6 ± 1.47 ^a,AB^
L	C_L_SM	18.2 ± 1.29 ^a,AB^	19.2 ± 0.77 ^a,A^	62.6 ± 4.67 ^a,AB^	14.4 ± 1.05 ^a,B^	48.2 ± 1.83 ^a,B^	19.2 ± 1.76 ^a,A^
	C_L_SS	17.8 ± 0.70 ^a,AB^	19.8 ± 0.84 ^b,AB^	62.4 ± 2.96 ^a,B^	14.0 ± 0.61 ^a,B^	48.4 ± 3.55 ^a,AB^	19.8 ± 0.91 ^b,A^
	SMF_L_24	26.2 ± 0.93 ^b,C^	20.4 ± 0.78 ^b,AB^	53.4 ± 5.23 ^a,A^	12.1 ± 0.89 ^a,A^	41.3 ± 3.74 ^a,A^	20.4 ± 1.23 ^b,A^
	SMF_L_48	15.9 ± 1.55 ^a,A^	19.0 ± 1.15 ^b,A^	65.1 ± 5.62 ^a,B^	14.8 ± 0.53 ^a,B^	50.3 ± 4.26 ^a,B^	19.0 ± 1.84 ^a,A^
	SSF_L_24	19.9 ± 1.00 ^a,B^	21.0 ± 0.65 ^b,B^	59.2 ± 4.67 ^a,AB^	12.5 ± 0.56 ^a,A^	46.6 ± 4.17 ^a,AB^	21.0 ± 1.30 ^b,A^
	SSF_L_48	19.8 ± 1.49 ^a,B^	21.2 ± 1.29 ^b,AB^	59.0 ± 4.82 ^a,AB^	12.6 ± 1.12 ^a,AB^	46.4 ± 2.86 ^a,AB^	21.2 ± 1.99 ^b,A^

C—control samples (non-fermented); L—lentils obtained from pure stands; LR—lentils obtained from relay incorporated with winter rye; SM—submerged conditions (lentils/water ratio 1:5, *w*/*w*); SS—submerged conditions (lentils/water ratio 1:1, *w*/*w*); SMF—fermented under submerged conditions; SSF—fermented under solid-state conditions; 24, 48—duration of fermentation (in hours). SFA—saturated fatty acids; MUFA—monounsaturated fatty acids; PUFA—polyunsaturated fatty acids. ^a,b^ Mean values denoted with different letters indicate significantly different values between the different lentil sample groups (LR and L) with the same treatment (SM, SF, SMF24, SMF48, SSF24, and SSF48); ^A–C^ mean values denoted with different letters indicate significantly different values between different treatments (SM, SF, SMF24, SMF48, SSF24, and SSF48) within the same group (LR or L). Results are statistically significant when *p* ≤ 0.05.

**Table 7:** Micro- and macro-element concentrations in lentil wholemeal samples.

	LR	L
	Macro-elements, mg/kg d.m. (dry matter)	
23 Na	7.12 ± 0.45 ^A^	21.0 ± 1.85 ^B^
24 Mg	676 ± 32.1 ^A^	726 ± 56.3 ^A^
39 K	4118 ± 115 ^A^	4925 ± 136 ^B^
44 Ca	510 ± 49.1 ^A^	747 ± 57.3 ^B^
	Essential micro-elements, mg/kg d.m. (dry matter)	
31 P	3406 ± 123 ^A^	3899 ± 142 ^B^
52 Cr	0.405 ± 0.39 ^A^	0.405 ± 0.028 ^A^
55 Mn	8.96 ± 0.52 ^A^	13.9 ± 0.93 ^B^
56 Fe	116 ± 9.35 ^B^	96.3 ± 7.41 ^A^
59 Co	0.059 ± 0.04 ^A^	0.064 ± 0.007 ^A^
60 Ni	1.43 ± 0.11 ^B^	0.893 ± 0.09 ^A^
63 Cu	7.29 ± 0.62 ^A^	7.12 ± 0.41 ^A^
66 Zn	22.0 ± 1.93 ^A^	24.6 ± 1.84 ^A^
78 Se	nd	0.014 ± 0.002 ^A^
	Non-essential micro-elements, mg/kg d.m. (dry matter)	
7 Li	nd	nd
9 Be	nd	nd
11 B	2.48 ± 0.12 ^A^	6.62 ± 0.58 ^B^
27 Al	88.3 ± 1.69 ^B^	25.3 ± 1.32 ^A^
47 Ti	3.19 ± 0.25 ^B^	0.767 ± 0.052 ^A^
51 V	nd	nd
71 Ga	nd	nd
75 As	0.019 ± 0.002 ^A^	0.017 ± 0.002 ^A^
85 Rb	7.22 ± 0.52 ^A^	6.33 ± 0.29 ^A^
88 Sr	2.65 ± 0.22 ^A^	3.02 ± 0.30 ^A^
95 Mo	2.09 ± 0.19 ^A^	1.84 ± 0.17 ^A^
105 Pd	nd	nd
107 Ag	nd	nd
111 Cd	nd	nd
118 Sn	nd	nd
121 Sb	nd	nd
133 Cs	nd	nd
137 Ba	0.865 ± 0.023 ^A^	1.49 ± 0.11 ^B^
201 Hg	nd	nd
208 Pb	0.177 ± 0.012 ^B^	0.036 ± 0.003 ^A^
115 In	nd	nd
195 Pt	nd	nd
205 Tl	nd	nd
209 Bi	nd	nd
238 U	nd	nd

L—lentils obtained from pure stands; LR—lentils obtained from relay incorporated with winter rye; ^A,B^ mean values denoted with different letters indicate significantly different values between the LR and L of lentil samples. Results are statistically significant when *p* ≤ 0.05. nd—not detected.

## Data Availability

The original contributions presented in the study are included in the article/supplementary material, further inquiries can be directed to the corresponding author.

## Supplementary Materials

The following supporting information can be downloaded at: https://www.mdpi.com/article/10.3390/foods13081249/s1. Table S1. Significance of analysed factors (different growing conditions of lentils, different fermentation conditions, and different durations of fermentation) and their interaction in lentil wholemeal pH, chromaticity, and lactic acid bacteria (LAB) viable counts. Table S2. Pearson correlations between the pH, chromaticity, and lactic acid bacteria (LAB) viable counts in lentil wholemeal samples. Table S3. Significance of analysed factors (different growing conditions of lentils, different fermentation conditions, and different durations of fermentation) and their interaction in lentil wholemeal free amino acids (FAA) and γ-aminobutyric acid (GABA). Table S4. Pearson correlations between the free amino acids (FAA) and γ-aminobutyric acid (GABA) and pH and lactic acid bacteria (LAB) viable counts in lentil whole samples. Table S5. Pearson correlations between biogenic amines (BA) and free amino acids (FAA), γ-aminobutyric acid (GABA), pH, and lactic acid bacteria (LAB) viable counts in lentil whole samples. Table S6. Significance of analysed factors (different growing conditions of lentils, different fermentation conditions, and different durations of fermentation) and their interaction in biogenic amine (BA) lentil wholemeal concentration. Table S7. Pearson correlations between fatty acids (FA) and pH and lactic acid bacteria (LAB) viable counts in lentil wholemeal samples. Table S8. Significance of analysed factors (different growing conditions of lentils, different fermentation conditions, and different durations of fermentation) and their interaction in fatty acid (FA) lentil wholemeal composition. Table S9. Volatile compound (VC) profile (% of the total volatile compounds content) in lentil whole samples. Table S10. Pearson correlations between volatiles (VCs) and pH and lactic acid bacteria (LAB) viable counts, as well as biogenic amines (BAs) and fatty acid (FA) composition, in lentil whole samples. Table S11. Pearson correlations between volatiles and free amino acids (FAA) and γ-aminobutyric acid (GABA). Table S12. Significance of analysed factors (different growing conditions of lentils, different fermentation conditions, and different durations of fermentation) and their interaction in volatile compound (VC) lentil wholemeal composition. Figure S1. Image of the relay intercropped lentil with winter rye (LR).

## Abbreviations

9-octadecenoic acid (C18:1); α-linolenic acid (C18:3 α); analysis of variance (ANOVA); biogenic amines (BA); brightness/darkness (L*/−L*); cadaverine (CAD); cis-11-eicosenoic acid (C20:1); colony-forming units (CFU); de Man–Rogosa–Sharpe (MRS) agar; diode array detector (DAD); dry matter (d.m.); eicosanoic acid (C20:1); fatty acid methyl esters (FAME); fatty acids (FAs); folic acid (vitamin B9); Food and Agriculture Organization (FAO); Food and Drug Administration (FDA); free amino acids (FAA); γ-aminobutyric acid (GABA); gas chromatographer(ry)/mass spectrometer, gas chromatography(/ph) mass spectrometry(/ter) (GC-MS); high-performance liquid chromatography (HPLC); histamine (HIS); inductively coupled plasma mass spectrometry (ICP-MS); internal diameter (ID); internal standard (ISTD); lactic acid bacteria (LAB); lentils grown in pure stands (L); lentils grown in relay intercropped with winter rye (LR); linoleic acid (C18:2); monounsaturated fatty acids (MUFA); National Bureau of Standards (NBS); niacin (vitamin B3); no observed adverse effect level (NOAEL); non-fermented (NF); palmitic acid (C16:0); pantothenic acid (vitamin B5); phenylethylamine (PHE); polyunsaturated fatty acids (PUFA); putrescine (PUT); pyridoxine (vitamin B6); redness/greenness (a*/−a*); riboflavin (vitamin B2); saturated fatty acids (SFA); solid-phase microextraction (SPME); solid-state (SS); solid-state fermentation (SSF); spermidine (SPRMD); spermine (SPRM); standard error (SE); stearic acid (C18:0); submerged (SM); submerged fermentation (SMF); tetradecanoic acid (C14:0); thiamine (vitamin B1); tryptamine (TRIP); tyramine (TYR); United Nations (UN); United States of America (USA); volatile compounds (VCs); volume by volume (*v*/*v*); wavelength (λ); weight by weight (*w*/*w*); World Health Organization (WHO); yellowness/blueness (b*/−b*).
