# Supplementary material for: Changes in Chemical Composition of Lentils, Including Gamma-Aminobutyric Acid and Volatile Compound Formation during Submerged and Solid-State Fermentation with Pediococcus acidilactici
Source: Foods. 2024 Apr 19;13(8):1249. doi: 10.3390/foods13081249 (PMC11049090; doi:10.3390/foods13081249)
Supplement: Supplementary file 1 [file foods-13-01249-s001.zip › foods-2956862-supplementary.pdf]

Supplementary information

Table S1. Significance of analysed factors (different growing conditions of lentils, different fermentation conditions and different duration of fermentation) and their interaction in lentil wholemeal pH, chromaticity and lactic acid bacteria (LAB) viable counts.

Factor	Dependent variable	p
Growing conditions	pH	**<0.001**
	L*	**<0.001**
	a*	**<0.001**
	b*	**<0.001**
	LAB count	0.093
Type of fermentation	pH	**<0.001**
	L*	**<0.001**
	a*	**<0.001**
	b*	**<0.001**
	LAB count	**0.005**
Fermentation duration	pH	**<0.001**
	L*	**<0.001**
	a*	**<0.001**
	b*	**<0.001**
	LAB count	**<0.001**
Growing conditions * Type of fermentation	pH	**<0.001**
	L*	**<0.001**
	a*	**0.005**
	b*	**0.002**
	LAB count	0.682
Growing conditions * Fermentation duration	pH	0.312
	L*	**<0.001**
	a*	**<0.001**
	b*	**<0.001**
	LAB count	**0.030**
Type of fermentation * Fermentation duration	pH	**<0.001**
	L*	**<0.001**
	a*	**<0.001**
	b*	**<0.001**
	LAB count	0.522
Growing conditions * Type of fermentation * Fermentation duration	pH	**<0.001**
	L*	**<0.001**
	a*	0.829
	b*	**<0.001**
	LAB count	0.522

L* – lightness; a* – redness (−a* greenness); b* – yellowness (−b* blueness); LAB – lactic acid bacteria, p – significance. The

influence of analysed factors and their interaction are significant, when $p \leq 0.05$. In **Bold** letters are marked significant values.

Table S2. Pearson correlations between the pH, chromaticity and lactic acid bacteria (LAB) viable counts in lentil wholemeal samples.

		pH	L*	a*	b*	LAB count
pH	Pearson Correlation	1	**0.763****	**-0.679****	**0.839****	**-0.886****
	Sig. (2-tailed)		<0.001	<0.001	<0.001	<0.001
L*	Pearson Correlation	**0.763****	1	**-0.871****	**0.955****	**-0.710****
	Sig. (2-tailed)	<0.001		<0.001	<0.001	<0.001
a*	Pearson Correlation	**-0.679****	**-0.871****	1	**-0.797****	**0.552****
	Sig. (2-tailed)	<0.001	<0.001		<0.001	<0.001
b*	Pearson Correlation	**0.839****	**0.955****	**-0.797****	1	**-0.799****
	Sig. (2-tailed)	<0.001	<0.001	<0.001		<0.001
LAB count	Pearson Correlation	**-0.886****	**-0.710****	**0.552****	**-0.799****	1
	Sig. (2-tailed)	<0.001	<0.001	<0.001	<0.001	

L* – lightness; a* – redness (−a* greenness); b* – yellowness (−b* blueness); LAB – lactic acid bacteria; ** Correlation is significant at the 0.01 level (2-tailed). * Correlation is significant at the 0.05 level (2-tailed). In **Bold** letters are marked significant values.

Table S3. Significance of analysed factors (different growing conditions of lentils, different fermentation conditions and different duration of fermentation) and their interaction in lentil wholemeal free amino acids (FAA) and γ-aminobutyric acid (GABA).

Factor	Dependent variable	p
Growing conditions	Arginine	**<0.001**
	Glutamine	**<0.001**
	Asparagine	**<0.001**
	Glutamic acid	**0.008**
	Serine	**<0.001**
	Aspartic acid	**<0.001**
	Threonine	**<0.001**
	Glycine	0.259
	Alanine	0.683
	GABA	**<0.001**
	Proline	**<0.001**
	Valine	**<0.001**
	Methionine	**<0.001**
	Phenylalanine	**0.006**
	Leucine/Isoleucine	**<0.001**
	Lysine	**<0.001**

	histidine	0.363
	Tyrosine	**0.048**
Type of fermentation	Arginine	**<0.001**
	Glutamine	**<0.001**
	Asparagine	**<0.001**
	Glutamic acid	**0.002**
	Serine	**<0.001**
	Aspartic acid	**<0.001**
	Threonine	**<0.001**
	Glycine	**<0.001**
	Alanine	**<0.001**
	GABA	**<0.001**
	Proline	**<0.001**
	Valine	**<0.001**
	Methionine	**0.032**
	Phenylalanine	**<0.001**
	Leucine/Isoleucine	**<0.001**
	Lysine	**<0.001**
	histidine	**<0.001**
	Tyrosine	**<0.001**
Fermentation duration	Arginine	**<0.001**
	Glutamine	**<0.001**
	Asparagine	0.197
	Glutamic acid	**<0.001**
	Serine	**<0.001**
	Aspartic acid	**<0.001**
	Threonine	**<0.001**
	Glycine	**<0.001**
	Alanine	**0.008**
	GABA	**0.042**
	Proline	**<0.001**
	Valine	**<0.001**
	Methionine	**<0.001**
	Phenylalanine	**<0.001**
	Leucine/Isoleucine	**<0.001**
	Lysine	**<0.001**
	histidine	1.000
	Tyrosine	**<0.001**
Growing conditions * Type of fermentation	Arginine	**<0.001**
	Glutamine	**<0.001**
	Asparagine	**0.003**
	Glutamic acid	0.532
	Serine	**<0.001**
	Aspartic acid	0.156
	Threonine	**0.003**
	Glycine	**<0.001**

	Alanine	**<0.001**
	GABA	**<0.001**
	Proline	0.275
	Valine	0.310
	Methionine	0.912
	Phenylalanine	0.192
	Leucine/Isoleucine	**0.039**
	Lysine	**0.042**
	histidine	**0.007**
	Tyrosine	0.668
Growing conditions * Fermentation duration	Arginine	**<0.001**
	Glutamine	**<0.001**
	Asparagine	0.618
	Glutamic acid	0.446
	Serine	**0.002**
	Aspartic acid	0.150
	Threonine	0.265
	Glycine	0.891
	Alanine	0.273
	GABA	**0.003**
	Proline	0.200
	Valine	**0.006**
	Methionine	0.075
	Phenylalanine	**<0.001**
	Leucine/Isoleucine	**<0.001**
	Lysine	**<0.001**
	histidine	**0.013**
	Tyrosine	**<0.001**
Type of fermentation * Fermentation duration	Arginine	**<0.001**
	Glutamine	**<0.001**
	Asparagine	0.391
	Glutamic acid	**0.003**
	Serine	**<0.001**
	Aspartic acid	**<0.001**
	Threonine	**0.047**
	Glycine	**0.005**
	Alanine	0.116
	GABA	**0.017**
	Proline	0.919
	Valine	**<0.001**
	Methionine	**<0.001**
	Phenylalanine	**0.004**
	Leucine/Isoleucine	**<0.001**
	Lysine	0.175
	histidine	**<0.001**
	Tyrosine	**<0.001**

Growing conditions * Type of fermentation * Fermentation duration	Arginine	**<0.001**
	Glutamine	**<0.001**
	Asparagine	0.274
	Glutamic acid	0.073
	Serine	0.383
	Aspartic acid	0.062
	Threonine	0.553
	Glycine	0.683
	Alanine	0.637
	GABA	**<0.001**
	Proline	0.919
	Valine	0.672
	Methionine	0.137
	Phenylalanine	0.219
	Leucine/Isoleucine	0.415
	Lysine	0.407
	histidine	0.230
	Tyrosine	**0.004**

The influence of analysed factors and their interaction are significant when p ≤ 0.05. In **Bold** letters are marked significant values.

Table S4. Pearson correlations between the free amino acids (FAA) and γ-aminobutyric acid (GABA) and pH and lactic acid bacteria (LAB) viable counts in lentil whole samples.

		pH	LAB count
Arginine	Pearson Correlation	**0.784****	**-0.892****
	Sig. (2-tailed)	<0.001	<0.001
Glutamine	Pearson Correlation	**0.479****	**-0.593****
	Sig. (2-tailed)	0.003	<0.001
Asparagine	Pearson Correlation	0.103	-0.232
	Sig. (2-tailed)	0.548	0.173
Glutamic acid	Pearson Correlation	**-0.828****	**0.869****
	Sig. (2-tailed)	<0.001	<0.001
Serine	Pearson Correlation	-0.010	-0.136
	Sig. (2-tailed)	0.952	0.429
Aspartic acid	Pearson Correlation	**-0.758****	**0.794****
	Sig. (2-tailed)	<0.001	<0.001
Threonine	Pearson Correlation	**-0.670****	**0.609****
	Sig. (2-tailed)	<0.001	<0.001
Glycine	Pearson Correlation	**-0.431****	**0.369***
	Sig. (2-tailed)	0.009	0.027
Alanine	Pearson Correlation	**-0.360***	**0.329***
	Sig. (2-tailed)	0.031	0.050
GABA	Pearson Correlation	-0.032	0.049
	Sig. (2-tailed)	0.851	0.778
Proline	Pearson Correlation	**-0.608****	**0.472****

	Sig. (2-tailed)	<0.001	0.004
Valine	Pearson Correlation	**-0.709****	**0.693****
	Sig. (2-tailed)	<0.001	<0.001
Methionine	Pearson Correlation	-0.222	0.122
	Sig. (2-tailed)	0.194	0.477
Phenylalanine	Pearson Correlation	**-0.740****	**0.714****
	Sig. (2-tailed)	<0.001	<0.001
Leucine/Isoleucine	Pearson Correlation	**-0.808****	**0.783****
	Sig. (2-tailed)	<0.001	<0.001
Lysine	Pearson Correlation	**-0.661****	**0.537****
	Sig. (2-tailed)	<0.001	<0.001
Histidine	Pearson Correlation	0.204	-0.314
	Sig. (2-tailed)	0.232	0.062
Tyrosine	Pearson Correlation	**-0.442****	**0.433****
	Sig. (2-tailed)	0.007	0.008

LAB – lactic acid bacteria; ** Correlation is significant at the 0.01 level (2-tailed). * Correlation is significant at the 0.05 level (2-tailed). In **Bold** letters are marked significant values.

Table S5. Pearson correlations between biogenic amines (BA) and free amino acids (FAA), γ-aminobutyric acid (GABA), pH and lactic acid bacteria (LAB) viable counts in lentil whole samples.

		PHE	PUT	SPRMD	SPRM
pH	Pearson Correlation	-0.015	**-0.719****	0.178	-0.243
	Sig. (2-tailed)	0.931	<0.001	0.299	0.154
LAB count	Pearson Correlation	0.219	**0.761****	-0.008	**0.331***
	Sig. (2-tailed)	0.199	<0.001	0.961	0.049
Arginine	Pearson Correlation	-0.214	**-0.686****	0.235	-0.152
	Sig. (2-tailed)	0.211	<0.001	0.168	0.376
Glutamine	Pearson Correlation	**-0.481****	**-0.341***	0.271	**0.354***
	Sig. (2-tailed)	0.003	0.042	0.110	0.034
Asparagine	Pearson Correlation	**-0.405***	0.268	-0.073	-0.002
	Sig. (2-tailed)	0.014	0.114	0.671	0.992
Glutamic acid	Pearson Correlation	0.069	**0.734****	-0.020	**0.461****
	Sig. (2-tailed)	0.690	<0.001	0.908	0.005
Serine	Pearson Correlation	**-0.410***	-0.085	0.105	**0.430****
	Sig. (2-tailed)	0.013	0.621	0.541	0.009
Aspartic acid	Pearson Correlation	0.046	**0.881****	-0.056	0.229
	Sig. (2-tailed)	0.791	<0.001	0.745	0.180
Threonine	Pearson Correlation	-0.250	**0.594****	0.041	**0.563****
	Sig. (2-tailed)	0.142	<0.001	0.814	<0.001
Glycine	Pearson Correlation	**-0.343***	**0.349***	0.315	**0.644****
	Sig. (2-tailed)	0.041	0.037	0.062	<0.001
Alanine	Pearson Correlation	-0.237	0.312	0.314	**0.787****
	Sig. (2-tailed)	0.164	0.064	0.062	<0.001
GABA	Pearson Correlation	-0.329	-0.066	**0.424****	**0.566****
	Sig. (2-tailed)	0.050	0.700	0.010	<0.001
Proline	Pearson Correlation	**-0.342***	**0.468****	0.023	**0.604****
	Sig. (2-tailed)	0.041	0.004	0.895	<0.001
Valine	Pearson Correlation	-0.097	**0.614****	0.065	**0.520****
	Sig. (2-tailed)	0.575	<0.001	0.704	0.001

Methionine	Pearson Correlation	-0.112	0.319	**0.356***	0.211
	Sig. (2-tailed)	0.516	0.058	0.033	0.217
Phenylalanine	Pearson Correlation	-0.046	**0.639****	0.067	**0.610****
	Sig. (2-tailed)	0.792	<0.001	0.697	<0.001
Leucine/Isoleucine	Pearson Correlation	-0.016	**0.697****	0.027	**0.425****
	Sig. (2-tailed)	0.925	<0.001	0.876	0.010
Lysine	Pearson Correlation	-0.298	**0.583****	0.052	**0.575****
	Sig. (2-tailed)	0.077	<0.001	0.763	<0.001
histidine	Pearson Correlation	**-0.339***	-0.181	0.316	**0.668****
	Sig. (2-tailed)	0.043	0.290	0.061	<0.001
Tyrosine	Pearson Correlation	0.043	0.313	**0.534****	**0.466****
	Sig. (2-tailed)	0.804	0.063	<0.001	0.004

TRIP – tryptamine; PHE – phenylethylamine; PUT – putrescine; CAD – cadaverine; HIS – histamine; TYR – tyramine; SPRMD – spermidine; SPRM – spermine; BA – biogenic amines; LAB – lactic acid bacteria. ** Correlation is significant at the 0.01 level (2-tailed). * Correlation is significant at the 0.05 level (2-tailed). In **Bold** letters are marked significant values.

Table S6. Significance of analysed factors (different growing conditions of lentils, different fermentation conditions and different duration of fermentation) and their interaction in biogenic amine (BA) lentil wholemeal concentration.

Factor	Dependent variable	p
Growing conditions	PHE	**<0.001**
	PUT	**<0.001**
	SPRMD	**<0.001**
	SPRM	0.170
Type of fermentation	PHE	**<0.001**
	PUT	**<0.001**
	SPRMD	0.066
	SPRM	**<0.001**
Fermentation duration	PHE	**<0.001**
	PUT	**<0.001**
	SPRMD	**<0.001**
	SPRM	0.249
Growing conditions * Type of fermentation	PHE	**<0.001**
	PUT	**0.019**
	SPRMD	**<0.001**
	SPRM	**<0.001**
Growing conditions * Fermentation duration	PHE	**<0.001**
	PUT	0.102
	SPRMD	**<0.001**
	SPRM	**0.040**
Type of fermentation * Fermentation duration	PHE	**<0.001**
	PUT	0.117
	SPRMD	**<0.001**
	SPRM	0.220
Growing conditions * Type of fermentation * Fermentation duration	PHE	**<0.001**
	PUT	0.154
	SPRMD	**<0.001**
	SPRM	0.937

p – significance; TRIP – tryptamine; PHE – phenylethylamine; PUT – putrescine; CAD – cadaverine; HIS – histamine; TYR – tyramine; SPRMD – spermidine; SPRM – spermine; BA – biogenic amines. The influence of analysed factors and their interaction are significant when $p \leq 0.05$. In **Bold** letters are marked significant values.

Table S7. Pearson correlations between fatty acids (FA) and pH and lactic acid bacteria (LAB) viable counts in lentil wholemeal samples.

		pH	LAB count
C14:0	Pearson Correlation	0.159	0.148
	Sig. (2-tailed)	0.355	0.388
C16:0	Pearson Correlation	0.212	-0.028
	Sig. (2-tailed)	0.214	0.873
C18:0	Pearson Correlation	-0.112	**0.363***
	Sig. (2-tailed)	0.517	0.030
C18:1	Pearson Correlation	-0.041	0.014
	Sig. (2-tailed)	0.811	0.937
C18:2	Pearson Correlation	-0.069	0.026
	Sig. (2-tailed)	0.689	0.879
C18:3 α	Pearson Correlation	0.031	-0.032
	Sig. (2-tailed)	0.857	0.855
C20:0	Pearson Correlation	-0.235	0.249
	Sig. (2-tailed)	0.168	0.142
C20:1	Pearson Correlation	-0.240	0.088
	Sig. (2-tailed)	0.159	0.608

C14:0 - tetradecanoic acid; C16:0 - palmitic acid; C18:0 - stearic acid; C18:1 - 9-octadecenoic acid; C18:2 - linoleic acid; C18:3 α - α-linolenic acid; C20:0 - eicosanoic acid; C20:1 - cis-11-eicosenoic acid. ** Correlation is significant at the 0.01 level (2-tailed). * Correlation is significant at the 0.05 level (2-tailed). In **Bold** letters are marked significant values.

Table S8. Significance of analysed factors (different growing conditions of lentils, different fermentation conditions and different duration of fermentation) and their interaction in fatty acid (FA) lentil wholemeal composition.

Factor	Dependent variable	p
Growing conditions	C14:0	**<0.001**
	C16:0	0.325
	C18:0	0.824
	C18:1	**<0.001**
	C18:2	**0.015**
	C18:3 α	**<0.001**
	C20:0	**<0.001**
	C20:1	**<0.001**
Type of fermentation	C14:0	**<0.001**
	C16:0	**<0.001**
	C18:0	0.364
	C18:1	**<0.001**
	C18:2	0.398
	C18:3 α	**<0.001**
	C20:0	**<0.001**
	C20:1	**<0.001**
Fermentation duration	C14:0	**<0.001**
	C16:0	**<0.001**
	C18:0	0.276
	C18:1	0.881
	C18:2	0.125
	C18:3 α	**0.005**
	C20:0	**<0.001**
	C20:1	**<0.001**
Growing conditions * Type of fermentation	C14:0	**<0.001**
	C16:0	**0.022**
	C18:0	**<0.001**
	C18:1	0.376
	C18:2	0.113
	C18:3 α	**0.031**
	C20:0	**<0.001**
	C20:1	**<0.001**
Growing conditions * Fermentation duration	C14:0	**<0.001**
	C16:0	**<0.001**
	C18:0	**<0.001**
	C18:1	0.552
	C18:2	**0.003**
	C18:3 α	**0.012**
	C20:0	**<0.001**
	C20:1	**<0.001**
	C14:0	**<0.001**

Type of fermentation * Fermentation duration	C16:0	**0.005**
	C18:0	**<0.001**
	C18:1	0.376
	C18:2	0.102
	C18:3 α	**0.005**
	C20:0	**<0.001**
	C20:1	**<0.001**
Growing conditions * Type of fermentation * Fermentation duration	C14:0	**<0.001**
	C16:0	**<0.001**
	C18:0	**<0.001**
	C18:1	**0.020**
	C18:2	**0.002**
	C18:3 α	**0.012**
	C20:0	**<0.001**
	C20:1	**<0.001**

p – significance; C14:0 - tetradecanoic acid; C16:0 - palmitic acid; C18:0 - stearic acid; C18:1 - 9-octadecenoic acid; C18:2 - linoleic acid; C18:3 α - α-linolenic acid; C20:0 - eicosanoic acid; C20:1 - cis-11-eicosenoic acid. The influence of analysed factors and their interaction are significant, when $p \leq 0.05$. In **Bold** letters are marked significant values.

Table S9. Volatile compound (VC) profile (% from the total volatile compounds content) in lentil whole samples.

Name	LR						L					
	$C_{LR}SM$	$C_{LR}SS$	$SMF_{LR}24$	$SMF_{LR}48$	$SSF_{LR}24$	$SSF_{LR}48$	$C_{L}SM$	$C_{L}SS$	$SMF_{L}24$	$SMF_{L}48$	$SSF_{L}24$	$SSF_{L}48$
Hexanal	23.82 $\pm2.25^{a,D}$	15.50 $\pm1.51^{b,B}$	nd	nd	18.71 $\pm1.38^{b,C}$	9.57 $\pm0.47^{b,A}$	30.77 $\pm1.74^{b,D}$	8.70 $\pm0.656^{a,B}$	7.95 $\pm0.262^{a,B}$	nd	10.52 $\pm0.56^{a,C}$	3.12 $\pm0.23^{a,A}$
2-Hexenal	4.89 $\pm0.48^{a,C}$	3.15 $\pm0.242^{a,B}$	nd	nd	0.546 $\pm0.029^{a,A}$	nd	11.16 $\pm0.49^{b,A}$	nd	nd	nd	nd	nd
1-Hexanol	35.03 $\pm1.33^{b,D}$	31.10 $\pm1.66^{b,C,D}$	5.44 $\pm0.368^{a,A}$	22.22 $\pm2.06^{b,B}$	28.54 $\pm1.19^{a,C}$	32.85 $\pm1.20^{b,D}$	21.41 $\pm1.46^{a,B}$	25.05 $\pm0.917^{a,C}$	26.72 $\pm1.25^{b,C}$	14.00 $\pm0.996^{a,A}$	32.17 $\pm2.14^{b,D}$	27.45 $\pm1.67^{a,C}$
Hept-2-enal	nd	nd	0.252 $\pm0.015^{a,A}$	nd	0.830 $\pm0.079^{a,B}$	1.20 $\pm0.107^{a,C}$	nd	nd	0.415 $\pm0.022^{b,A}$	nd	nd	nd
Benzaldehyde	nd	nd	0.629 $\pm0.06^{a,A}$	nd	3.53 $\pm0.276^{a,B}$	6.05 $\pm0.366^{a,C}$	nd	nd	nd	nd	5.52 $\pm0.433^{b,A}$	6.63 $\pm0.49^{a,B}$
Vinyl amyl carbinol	nd	nd	nd	nd	0.357 $\pm0.036^{a,A}$	nd	nd	nd	nd	nd	0.573 $\pm0.042^{b,B}$	0.462 $\pm0.024^{a,A}$
Hexanoic acid	4.75 $\pm0.311^{a,A}$	5.93 $\pm0.471^{a,B}$	5.58 $\pm0.328^{a,B}$	14.00 $\pm0.724^{b,E}$	9.13 $\pm0.396^{b,C}$	11.45 $\pm0.519^{b,D}$	5.74 $\pm0.479^{b,B}$	7.13 $\pm0.633^{a,C}$	4.98 $\pm0.294^{a,A,B}$	4.80 $\pm0.206^{a,A}$	4.63 $\pm0.144^{a,A}$	6.61 $\pm0.358^{a,C}$

Acetic acid hexyl ester	nd	nd	nd	nd	1.13 ±0.063 ^{b,A}	2.26 ±0.072 ^{b,B}	nd	nd	nd	nd	0.782 ±0.027 ^{a,A}	1.07 ±0.098 ^{a,B}
Oct-3-en-2-one	nd	nd	nd	nd	nd	0.544 ±0.042 ^{a,A}	nd	nd	nd	nd	0.562 ±0.05 ^{a,A}	0.499 ±0.031 ^{a,A}
Phenylacetaldehyde	0.14 ±0.013 ^{a,A}	nd	nd	nd	1.25 ±0.095 ^{b,B}	1.39 ±0.086 ^{a,B}	0.280 ±0.015 ^{b,A}	nd	1.83 ±0.085 ^{b,D}	nd	0.995 ±0.034 ^{a,B}	1.34 ±0.108 ^{a,C}
Oct-2-enal	0.402 ±0.031 ^{a,A}	0.590 ±0.044 ^{a,B}	0.936 ±0.065 ^{a,C}	nd	2.41 ±0.132 ^{b,E}	1.55 ±0.079 ^{b,D}	0.371 ±0.029 ^{a,A}	nd	1.74 ±0.173 ^{b,C}	nd	1.96 ±0.144 ^{a,C}	1.35 ±0.074 ^{a,B}
Octahydro-2,3'-bifuran	nd	nd	nd	nd	0.606 ±0.041 ^{a,A}	nd	nd	nd	nd	nd	0.879 ±0.081 ^{b,A}	nd
Nonanal	0.961 ±0.072 ^{a,A}	1.63 ±0.104 ^{a,B}	1.83 ±0.1 ^{a,C}	3.27 ±0.116 ^{b,D}	1.93 ±0.114 ^{b,C}	1.92 ±0.125 ^{a,C}	1.23 ±0.122 ^{b,A}	1.67 ±0.132 ^{a,B}	1.65 ±0.093 ^{a,B}	2.89 ±0.113 ^{a,C}	1.70 ±0.068 ^{a,B}	1.89 ±0.175 ^{a,B}
Phenethyl alcohol	nd	nd	nd	nd	2.18 ±0.117 ^{a,A}	5.58 ±0.282 ^{a,B}	nd	nd	3.70 ±0.368 ^{a,B}	8.08 ±0.788 ^{a,D}	2.85 ±0.194 ^{b,A}	5.44 ±0.198 ^{a,C}
2-ethylhexanoic acid	0.673 ±0.065 ^{a,A}	1.14 ±0.074 ^{a,B}	2.00 ±0.066 ^{a,D}	nd	1.91 ±0.183 ^{b,D}	1.50 ±0.058 ^{b,C}	0.945 ±0.065 ^{b,A}	2.74 ±0.186 ^{b,D}	2.06 ±0.202 ^{a,C}	3.27 ±0.099 ^{a,E}	1.28 ±0.121 ^{a,B}	0.81 ±0.073 ^{a,A}

Non-2-enal	2.86 ±0.167 ^{a,C}	1.86 ±0.106^{a,B}	1.07 ±0.091 ^{a,A}	nd	2.17 ±0.214 ^{b,B}	0.930 ±0.067 ^{b,A}	4.90 ±0.314 ^{b,D}	1.76 ±0.174^{a,C}	1.81 ±0.18 ^{b,C}	nd	0.955 ±0.053 ^{a,B}	0.651 ±0.047 ^{b,A}
Octanoic acid	0.876 ±0.041 ^{a,A}	1.81 ±0.115^{a,C}	5.48 ±0.548 ^{a,E}	3.96 ±0.292 ^{a,D}	3.80 ±0.312 ^{b,D}	1.52 ±0.084 ^{a,B}	1.41 ±0.131 ^{b,A}	4.71 ±0.193^{b,D}	5.40 ±0.325 ^{a,E}	4.47 ±0.433 ^{a,D}	2.77 ±0.108 ^{a,C}	1.88 ±0.165 ^{b,B}
Decanal	0.532 ±0.021 ^{b,A}	0.720 ±0.025^{a,B}	0.844 ±0.08 ^{a,BC}	nd	0.727 ±0.044 ^{a,B}	0.839 ±0.066 ^{b,C}	0.419 ±0.031 ^{a,A}	1.22 ±0.09 ^{b,E}	0.884 ±0.043 ^{a,D}	1.33 ±0.059 ^{a,E}	0.756 ±0.036 ^{a,C}	0.683 ±0.022 ^{a,B}
Nona-2,4-dienal	nd	nd	nd	nd	nd	0.323 ±0.032 ^{a,A}	nd	nd	nd	nd	nd	0.306 ±0.022 ^{a,A}
Dec-2-enal	nd	nd	nd	nd	0.201 ±0.015 ^{a,A}	nd	nd	nd	nd	nd	0.294 ±0.016 ^{b,B}	0.171 ±0.015 ^{a,A}
Nonanoic acid	0.510 ±0.038 ^{a,A}	2.05 ±0.186^{a,B}	2.61 ±0.22^{a,C}	nd	3.50 ±0.334 ^{a,D}	2.37 ±0.203 ^{b,BC}	0.789 ±0.079^{b,A}	5.33 ±0.258^{b,E}	2.53 ±0.245 ^{a,C}	5.05 ±0.213 ^{a,E}	3.55 ±0.153 ^{a,D}	1.83 ±0.18 ^{a,B}
Anethole	nd	nd	nd	nd	nd	nd	3.15 ±0.272 ^{a,C}	4.56 ±0.25 ^{a,D}	7.68 ±0.372 ^{a,F}	6.62 ±0.658 ^{a,E}	1.27 ±0.045 ^{a,A}	2.19 ±0.08 ^{a,B}
Tridecane	nd	nd	nd	nd	nd	nd	nd	nd	0.346 ±0.024 ^{a,B}	nd	nd	0.234 ±0.014 ^{a,A}
Undecanal	0.133 ±0.006 ^{a,A}	nd	nd	1.52 ±0.07 ^{a,C}	nd	0.451 ±0.019 ^{b,B}	nd	nd	nd	nd	0.090 ±0.006 ^{a,A}	0.240 ±0.015 ^{a,B}

2-methylpropanoic acid 2,2-dimethyl-1-(2-hydroxy-1-methylethyl)propyl ester	2.07 ±0.087 ^{a,A}	2.56 ±0.199^{a,BC}	4.72 ±0.162 ^{b,D}	nd	2.91 ±0.244 ^{a,C}	2.41 ±0.09 ^{b,B}	1.91 ±0.169 ^{a,B}	3.32 ±0.319^{b,C}	3.29 ±0.165 ^{a,C}	5.86 ±0.389 ^{a,D}	2.88 ±0.272 ^{a,C}	1.22 ±0.094 ^{a,A}
Eugenol	nd	nd	nd	nd	nd	nd	1.36 ±0.109 ^{a,A}	12.1 ±0.925^{a,E}	7.69 ±0.498 ^{a,D}	12.50 ±0.757 ^{a,E}	2.14 ±0.144 ^{a,B}	3.72 ±0.282 ^{a,C}
2-methylpropanoic acid 2-ethyl-3-hydroxyhexyl ester	2.53 ±0.119 ^{a,A}	2.88 ±0.197 ^{a,B}	5.28 ±0.419 ^{b,D}	4.91 ±0.329 ^{a,D}	3.57 ±0.252 ^{a,C}	3.42 ±0.228 ^{b,C}	2.57 ±0.218 ^{a,B}	5.57 ±0.38 ^{b,D}	4.33 ±0.257 ^{a,C}	9.02 ±0.829 ^{b,E}	4.17 ±0.375 ^{a,C}	1.63 ±0.1^{a,A}
β-Damascenone	2.67 ±0.189 ^{b,A}	3.52 ±0.351 ^{b,B}	6.17 ±0.607^{a,C}	31.39 ±2.488 ^{b,D}	2.54 ±0.13^{a,A}	3.92 ±0.379 ^{b,B}	1.88 ±0.095^{a,B}	1.60 ±0.12 ^{a,A}	5.61 ±0.308 ^{a,D}	7.86 ±0.274 ^{a,E}	2.65 ±0.25^{a,C}	2.97 ±0.205^{a,C}
1-Tetradecene	0.404 ±0.016 ^{a,A}	1.13 ±0.057 ^{a,C}	nd	nd	0.983 ±0.036 ^{a,B}	nd	nd	2.28 ±0.223^{b,A}	nd	nd	nd	nd
Tetradecane	0.333 ±0.011 ^{a,A}	0.430 ±0.03^{a,B}	1.14 ±0.037 ^{b,D}	3.08 ±0.129^{b,E}	0.443 ±0.037 ^{a,B}	0.939 ±0.047 ^{a,C}	0.389 ±0.034 ^{b,A}	0.635 ±0.03 ^{b,C}	0.852 ±0.084 ^{a,D}	2.38 ±0.113 ^{a,F}	0.542 ±0.035 ^{b,B}	1.09 ±0.062^{b,E}

Dodecanal	0.612 ±0.047 ^{a,A}	1.44 ±0.114 ^{a,C}	1.05 ±0.064 ^{a,B}	2.75 ±0.127 ^{b,D}	1.28 ±0.087 ^{b,C}	1.35 ±0.077 ^{b,C}	0.676 ±0.064 ^{a,A}	2.41 ±0.108 ^{b,F}	1.33 ±0.056 ^{b,D}	1.71 ±0.104 ^{a,E}	1.13 ±0.041 ^{a,C}	0.879 ±0.041 ^{a,B}
3-(2,2,6-Trimethyl-7-oxabicyclo[4.1.0]heptan-1-yl)prop-2-enal	0.63 ±0.031 ^{b,A}	1.06 ±0.096 ^{a,B}	1.53 ±0.078 ^{a,C}	6.20 ±0.437 ^{b,D}	1.24 ±0.106 ^{a,B}	1.55 ±0.146 ^{b,C}	0.369 ±0.034 ^{a,A}	1.52 ±0.047 ^{b,D}	1.43 ±0.101 ^{a,D}	3.51 ±0.235 ^{a,E}	1.18 ±0.042 ^{a,C}	0.811 ±0.061 ^{a,B}
Tridecanal	0.13 ±0.004 ^{a,A}	nd	nd	nd	nd	nd	nd	nd	nd	nd	nd	nd
((Butoxymethylethoxy)methylethoxy)-propanol	1.02 ±0.039 ^{a,A}	1.64 ±0.128 ^{a,B}	2.03 ±0.1 ^{a,C}	4.47 ±0.306 ^{a,E}	2.20 ±0.15 ^{b,C}	2.63 ±0.101 ^{b,D}	1.85 ±0.069 ^{b,C}	4.02 ±0.176 ^{b,E}	2.40 ±0.208 ^{b,D}	5.95 ±0.215 ^{b,F}	1.63 ±0.115 ^{a,B}	1.20 ±0.086 ^{a,A}
Hexadecane	0.354 ±0.035 ^{a,A}	0	0.900 ±0.085 ^{a,C}	2.20 ±0.193 ^{a,E}	0.640 ±0.032 ^{a,B}	1.04 ±0.049 ^{b,D}	nd	nd	0.936 ±0.06 ^{a,C}	nd	0.821 ±0.045 ^{b,B}	0.521 ±0.034 ^{a,A}
Tetradecanal	0.628 ±0.061 ^{a,A}	0.800 ±0.043 ^{b,B}	1.68 ±0.11 ^{a,C}	nd	nd	nd	0.543 ±0.034 ^{a,B}	0.653 ±0.058 ^{a,C}	nd	nd	0.462 ±0.044 ^{aA}	0.881 ±0.087 ^{a,D}
Tridecanedial	0.120 ±0.01 ^{b,B}	0.090 ±0.007 ^{a,A}	nd	nd	nd	nd	0.068 ±0.006 ^{a,A}	nd	0.247 ±0.009 ^{a,B}	nd	nd	0.310 ±0.018 ^{a,C}

Pentadecanal	6.03 ±0.582 ^{b,B}	7.31 ±0.245^{b,C}	23.9 ±1.085 ^{b,D}	nd	0.581 ±0.027 ^{a,A}	nd	2.77 ±0.096^{a,C}	1.45 ±0.087^{a,B}	1.19 ±0.101 ^{a,A}	nd	3.42 ±0.232 ^{b,D}	10.5 ±0.454^{a,E}
cis,cis-7,10,-Hexadecadienal	0.069 ±0.003 ^{a,A}	nd	0.348 ±0.028^{a,B}	nd	nd	nd	nd	nd	nd	nd	nd	0.195 ±0.014 ^{a,A}
7-Hexadecenal	nd	nd	0.864 ±0.072^{a,A}	nd	nd	nd	nd	nd	nd	nd	nd	nd
11-Hexadecyn-1-ol	4.03 ±0.369 ^{b,A}	5.71 ±0.525 ^{b,B}	11.02 ±0.714^{a,C}	nd	nd	nd	1.26 ±0.083^{a,B}	0.347 ±0.027^{a,A}	nd	nd	1.85 ±0.095 ^{a,C}	3.50 ±0.154 ^{a,D}
14-methyl-8-Hexadecenal	2.55 ±0.159 ^{b,A}	5.31 ±0.305 ^{b,B}	10.6 ±0.652^{b,C}	nd	nd	nd	1.72 ±0.125^{a,C}	0.730 ±0.036^{a,B}	0.522 ±0.024 ^{a,A}	nd	2.33 ±0.178 ^{a,D}	3.85 ±0.294^{a,E}
Methyl hexadecanoate	nd	nd	nd	nd	nd	nd	nd	nd	nd	nd	nd	0.4964±0.019 a,A
Hexadecanoic acid ethyl ester	0.131 ±0.009 ^{a,B}	0.550 ±0.026 ^{b,D}	1.50 ±0.113^{b,E}	nd	0.080 ±0.004 ^{a,A}	0.349 ±0.012 ^{a,C}	nd	0.366 ±0.034^{a,A}	0.387 ±0.038$^{a, A}$	0.605 ±0.043 ^{a,B}	0.428 ±0.04^{b,A}	0.793 ±0.077 ^{b,C}
Ethyl Oleate	0.047 ±0.004 ^{a,A}	nd	0.450 ±0.026^{a,B}	nd	nd	nd	nd	nd	nd	nd	0.150 ±0.007 ^{a,A}	0.428 ±0.018^{a,B}

C – control samples (non-fermented); L – lentil sobtained from pure stands; LR – lentils obtained from relay incorporated with winter rye; SM – submerged conditions (lentils/water ratio 1:5, w/w); SS – submerged conditions (lentils/water ratio 1:1, w/w); SMF – fermented under submerged conditions; SSF – fermented under solid-state conditions; 24, 48 – duration of fermentation (in hours).

[a–b] Mean values denoted with different letters indicate significantly different values between the different lentil sample groups (LR and L) with the same treatment (SM, SF, SMF24, SMF48, SSF24, SSF48); [A–E] Mean values denoted with different letters indicate significantly different values between different treatment (SM, SF, SMF24, SMF48, SSF24, SSF48) within the same group (LR or L). Results are statistically significant when $p \leq 0.05$. nd – not detected.

Table S10. Pearson correlations between volatiles (VC) and pH, lactic acid bacteria (LAB) viable counts, biogenic amines (BA) and fatty acid (FA) composition in lentil whole samples.

		pH	LAB count	PHE	PUT	SPR MD	SPR M	C14:0	C16:0	C18:0	C18:1	C18:2	C18:3 α	C20:0	C20:1
Hexanal	Pearson Correlation	**0.714****	**-0.737****	**-0.339***	**-0.694****	0.003	**-0.389***	-0.087	0.268	-0.095	0.087	-0.059	-0.057	-0.036	-0.006
	Sig. (2-tailed)	<0.001	<0.001	0.043	<0.001	0.987	0.019	0.613	0.114	0.582	0.612	0.733	0.740	0.837	0.973
2-Hexenal	Pearson Correlation	**0.610****	**-0.711****	-0.152	**-0.642****	-0.043	**-0.779****	-0.152	-0.060	-0.226	-0.008	0.087	0.157	-0.152	-0.153
	Sig. (2-tailed)	<0.001	<0.001	0.375	<0.001	0.805	<0.001	0.375	0.726	0.184	0.962	0.613	0.361	0.375	0.374
1-Hexanol	Pearson Correlation	0.212	-0.299	**-0.720****	-0.200	-0.081	0.301	0.060	**0.692****	0.310	**0.335***	**-0.336***	**-0.388***	0.284	0.259
	Sig. (2-tailed)	0.214	0.077	<0.001	0.242	0.640	0.074	0.730	<0.001	0.066	0.046	0.045	0.019	0.094	0.127
Hept-2-enal	Pearson Correlation	-0.162	**0.391***	0.022	-0.004	**0.419***	**0.403***	0.150	**0.339***	**0.444****	-0.179	-0.129	-0.108	**0.769****	-0.176
	Sig. (2-tailed)	0.345	0.018	0.899	0.981	0.011	0.015	0.383	0.043	0.007	0.297	0.452	0.529	<0.001	0.304
Benzaldehyde	Pearson Correlation	**-0.541****	**0.451****	-0.141	**0.331***	-0.165	**0.414***	-0.213	0.241	0.133	**0.336***	-0.103	**-0.451****	**0.482****	**0.422***
	Sig. (2-tailed)	<0.001	0.006	0.412	0.049	0.337	0.012	0.211	0.157	0.439	0.045	0.551	0.006	0.003	0.010
Vinyl amyl carbinol	Pearson Correlation	**-0.407***	0.292	-0.169	**0.381***	**-0.443****	0.319	-0.169	0.214	-0.025	**0.478****	-0.132	**-0.501****	-0.169	**0.670****

	Sig. (2-tailed)	0.014	0.084	0.324	0.022	0.007	0.058	0.324	0.209	0.884	0.003	0.444	0.002	0.324	<0.001
Hexanoic acid	Pearson Correlation	**-0.419***	**0.361***	-0.153	-0.125	**0.344***	0.199	-0.216	0.119	0.299	**-0.343***	0.112	**0.372***	**0.462****	-0.254
	Sig. (2-tailed)	0.011	0.031	0.373	0.467	0.040	0.244	0.207	0.491	0.076	0.040	0.516	0.025	0.005	0.135
Acetic acid hexyl ester	Pearson Correlation	**-0.438****	**0.420***	-0.188	0.143	0.135	**0.453****	-0.188	0.265	0.236	0.105	-0.084	-0.299	**0.786****	0.148
	Sig. (2-tailed)	0.007	0.011	0.272	0.405	0.433	0.006	0.272	0.119	0.166	0.542	0.625	0.077	<0.001	0.388
Oct-3-en-2-one	Pearson Correlation	**-0.523****	**0.365***	-0.173	**0.359***	**-0.374***	0.283	-0.173	0.199	0.082	**0.378***	-0.121	**-0.459****	**0.533****	**0.557****
	Sig. (2-tailed)	0.001	0.029	0.313	0.031	0.024	0.095	0.313	0.244	0.636	0.023	0.483	0.005	<0.001	<0.001
Phenylacetaldehyde	Pearson Correlation	-0.247	**0.426****	-0.270	**0.425****	0.028	0.291	**0.552****	**0.656****	**0.658****	**0.489****	**-0.558****	**-0.709****	**0.356***	0.176
	Sig. (2-tailed)	0.146	0.010	0.111	0.010	0.871	0.085	<0.001	<0.001	<0.001	0.002	<0.001	<0.001	0.033	0.304
Oct-2-enal	Pearson Correlation	-0.190	**0.390***	-0.002	0.274	-0.106	**0.401***	0.302	**0.597****	**0.472****	0.248	**-0.350***	**-0.532****	0.228	**0.381***
	Sig. (2-tailed)	0.268	0.019	0.992	0.106	0.540	0.015	0.074	<0.001	0.004	0.144	0.036	<0.001	0.182	0.022
Octahydro-2,3'-bifuran	Pearson Correlation	-0.209	0.167	-0.131	0.185	**-0.538****	0.313	-0.131	0.218	-0.072	0.213	-0.075	-0.317	-0.131	**0.806****
	Sig. (2-tailed)	0.221	0.329	0.445	0.279	<0.001	0.063	0.445	0.202	0.676	0.212	0.666	0.060	0.445	<0.001

Nonanal	Pearson Correlation	**-0.760****	**0.686****	-0.021	**0.533****	0.131	0.211	-0.110	-0.269	0.162	-0.160	0.258	**0.387***	0.025	-0.089
	Sig. (2-tailed)	<0.001	<0.001	0.902	<0.001	0.446	0.217	0.523	0.112	0.344	0.351	0.129	0.020	0.885	0.606
Phenethyl alcohol	Pearson Correlation	**-0.636****	**0.639****	-0.257	**0.799****	0.086	0.312	0.156	-0.067	0.221	**0.420***	-0.085	**-0.366***	**0.363***	0.060
	Sig. (2-tailed)	<0.001	<0.001	0.131	<0.001	0.619	0.064	0.365	0.698	0.196	0.011	0.623	0.028	0.030	0.730
2-ethylhexanoic acid	Pearson Correlation	0.025	0.152	0.161	**0.389***	0.292	**0.392***	0.184	-0.298	-0.144	0.213	0.175	-0.077	-0.009	-0.082
	Sig. (2-tailed)	0.887	0.375	0.348	0.019	0.084	0.018	0.283	0.078	0.403	0.211	0.306	0.656	0.961	0.633
Non-2-enal	Pearson Correlation	**0.801****	**-0.752****	-0.116	**-0.687****	0.074	**-0.549****	0.056	0.174	-0.099	0.109	-0.043	-0.031	-0.150	-0.145
	Sig. (2-tailed)	<0.001	<0.001	0.499	<0.001	0.670	<0.001	0.747	0.310	0.567	0.526	0.805	0.859	0.383	0.400
Octanoic acid	Pearson Correlation	-0.186	**0.443****	**0.439****	**0.454****	0.136	0.252	**0.421***	-0.130	0.154	-0.016	0.060	0.114	-0.309	-0.076
	Sig. (2-tailed)	0.277	0.007	0.007	0.005	0.428	0.138	0.011	0.448	0.370	0.928	0.730	0.510	0.067	0.660
Decanal	Pearson Correlation	0.040	0.083	0.091	**0.405***	0.225	**0.494****	0.126	-0.214	-0.165	**0.335***	0.136	-0.210	0.087	0.010
	Sig. (2-tailed)	0.818	0.629	0.597	0.014	0.187	0.002	0.465	0.209	0.336	0.046	0.430	0.219	0.615	0.955
Nona-2,4-dienal	Pearson Correlation	**-0.434****	**0.364***	-0.134	0.208	0.244	0.252	-0.134	0.141	0.229	0.213	-0.078	-0.272	**0.697****	-0.134

	Sig. (2-tailed)	0.008	0.029	0.436	0.224	0.151	0.137	0.436	0.413	0.180	0.213	0.652	0.108	<0.001	0.435
Dec-2-enal	Pearson Correlation	**-0.367***	0.272	-0.167	**0.337***	**-0.469****	**0.339***	-0.167	0.228	-0.034	**0.417***	-0.121	**-0.469****	-0.167	**0.722****
	Sig. (2-tailed)	0.028	0.109	0.330	0.044	0.004	0.043	0.330	0.182	0.842	0.011	0.483	0.004	0.330	<0.001
Nonanoic acid	Pearson Correlation	-0.098	0.158	0.022	**0.432****	0.132	**0.600****	0.007	-0.236	-0.232	0.327	0.159	-0.192	-0.024	0.196
	Sig. (2-tailed)	0.571	0.359	0.901	0.009	0.442	<0.001	0.969	0.166	0.173	0.051	0.353	0.263	0.889	0.252
Anethole	Pearson Correlation	0.038	0.061	-0.238	**0.542****	0.088	-0.094	**0.625****	-0.058	0.157	**0.592****	-0.258	**-0.379***	-0.238	-0.095
	Sig. (2-tailed)	0.825	0.722	0.162	<0.001	0.609	0.586	<0.001	0.738	0.362	<0.001	0.128	0.022	0.162	0.582
Tridecane	Pearson Correlation	-0.070	0.270	-0.131	**0.513****	0.091	0.020	**0.812****	**0.511****	**0.649****	**0.545****	**-0.594****	**-0.611****	-0.131	-0.132
	Sig. (2-tailed)	0.683	0.112	0.445	0.001	0.598	0.906	<0.001	0.001	<0.001	<0.001	<0.001	<0.001	0.445	0.444
Undecanal	Pearson Correlation	**-0.507****	**0.358***	-0.146	0.014	-0.051	-0.087	-0.146	0.018	0.218	-0.315	0.032	**0.361***	0.178	-0.081
	Sig. (2-tailed)	0.002	0.032	0.397	0.935	0.770	0.616	0.397	0.918	0.201	0.061	0.851	0.031	0.298	0.640
2-methylpropanoic acid 2,2-dimethyl-1-(2-hydroxy-	Pearson Correlation	0.035	0.159	**0.404***	**0.357***	0.078	0.228	0.111	**-0.372***	-0.212	0.037	0.298	0.062	-0.072	0.027
	Sig. (2-tailed)	0.841	0.353	0.015	0.033	0.653	0.181	0.519	0.025	0.215	0.832	0.078	0.718	0.678	0.878

1-methylethyl)propyl ester															
Eugenol	Pearson Correlation	-0.041	0.048	-0.216	0.507**	0.154	0.198	0.289	-0.286	-0.137	0.522**	-0.022	-0.235	-0.216	-0.075
	Sig. (2-tailed)	0.814	0.779	0.207	0.002	0.370	0.247	0.087	0.091	0.427	0.001	0.899	0.167	0.207	0.662
2-methylpropanoic acid 2-ethyl-3-hydroxyhexyl ester	Pearson Correlation	-0.321	0.371*	0.183	0.487**	0.033	0.179	0.030	-0.514**	-0.187	-0.053	0.364*	0.322	-0.115	0.004
	Sig. (2-tailed)	0.056	0.026	0.286	0.003	0.848	0.296	0.863	0.001	0.274	0.757	0.029	0.055	0.504	0.982
β-Damascenone	Pearson Correlation	-0.480**	0.404*	0.005	0.137	-0.044	-0.145	-0.017	-0.095	0.214	-0.374*	0.095	0.479**	-0.081	-0.130
	Sig. (2-tailed)	0.003	0.015	0.975	0.425	0.797	0.398	0.924	0.581	0.211	0.025	0.581	0.003	0.638	0.448
1-Tetradecene	Pearson Correlation	0.558**	-0.622**	-0.174	-0.605**	0.151	-0.548**	-0.174	0.042	-0.103	-0.069	0.065	0.144	-0.174	-0.174
	Sig. (2-tailed)	<0.001	<0.001	0.310	<0.001	0.381	<0.001	0.311	0.808	0.550	0.688	0.706	0.401	0.310	0.310
Tetradecane	Pearson Correlation	-0.698**	0.611**	0.044	0.469**	0.037	-0.046	-0.060	-0.375*	0.087	-0.214	0.224	0.422*	-0.030	-0.175
	Sig. (2-tailed)	<0.001	<0.001	0.797	0.004	0.828	0.790	0.726	0.024	0.614	0.210	0.190	0.010	0.864	0.307
Dodecanal	Pearson Correlation	-0.281	0.209	-0.159	0.157	0.229	0.344*	-0.023	-0.115	0.073	-0.144	0.127	0.336*	-0.016	-0.124

	Sig. (2-tailed)	0.097	0.222	0.356	0.360	0.178	0.040	0.894	0.503	0.673	0.402	0.461	0.045	0.928	0.472
3-(2,2,6-Trimethyl-7-oxabicyclo[4.1.0]heptan-1-yl)prop-2-enal	Pearson Correlation	**-0.577****	**0.493****	-0.042	0.304	0.020	0.030	-0.062	-0.244	0.119	-0.311	0.184	**0.474****	-0.038	-0.111
	Sig. (2-tailed)	<0.001	0.002	0.807	0.072	0.906	0.862	0.720	0.152	0.491	0.065	0.281	0.003	0.824	0.521
Tridecanal	Pearson Correlation	**0.407***	**-0.390***	-0.091	**-0.413***	-0.153	-0.173	-0.091	0.049	-0.194	-0.094	0.056	0.112	-0.091	-0.091
	Sig. (2-tailed)	0.014	0.019	0.599	0.012	0.372	0.313	0.599	0.777	0.257	0.584	0.745	0.517	0.599	0.598
((Butoxymethylethoxy)methylethoxy)-propanol	Pearson Correlation	**-0.398***	**0.344***	-0.117	**0.392***	0.237	0.139	-0.037	**-0.476****	-0.103	-0.057	0.282	**0.349***	0.010	-0.201
	Sig. (2-tailed)	0.016	0.040	0.498	0.018	0.164	0.420	0.830	0.003	0.549	0.742	0.095	0.037	0.952	0.240
Hexadecane	Pearson Correlation	**-0.554****	**0.611****	0.140	0.190	-0.176	0.038	0.157	0.244	**0.449****	-0.292	-0.106	0.182	0.211	0.100
	Sig. (2-tailed)	<0.001	<0.001	0.416	0.267	0.304	0.828	0.361	0.152	0.006	0.084	0.537	0.289	0.217	0.561
Tetradecanal	Pearson Correlation	0.283	-0.256	**0.741****	-0.278	-0.117	-0.137	-0.286	-0.251	**-0.388***	-0.169	**0.359***	0.235	-0.286	-0.005
	Sig. (2-tailed)	0.095	0.132	<0.001	0.101	0.498	0.427	0.091	0.139	0.019	0.323	0.032	0.168	0.091	0.978
Tridecanedial	Pearson Correlation	0.096	-0.015	-0.204	0.250	0.089	-0.113	**0.521****	**0.457****	**0.459****	**0.519****	**-0.469****	**-0.514****	-0.204	-0.204
	Sig. (2-tailed)	0.579	0.932	0.233	0.142	0.606	0.512	0.001	0.005	0.005	0.001	0.004	0.001	0.233	0.232

Pentadecanal	Pearson Correlation	0.078	0.043	**0.874** **	-0.102	-0.090	-0.104	-0.162	-0.215	-0.205	-0.291	0.304	0.245	-0.217	-0.061
	Sig. (2-tailed)	0.652	0.803	<0.001	0.556	0.602	0.546	0.344	0.209	0.230	0.085	0.071	0.149	0.204	0.724
cis,cis-7,10,-Hexadecadienal	Pearson Correlation	-0.122	0.254	**0.852** **	0.045	-0.015	-0.070	-0.145	-0.275	-0.173	-0.206	0.299	0.201	-0.145	-0.146
	Sig. (2-tailed)	0.480	0.136	<0.001	0.794	0.929	0.684	0.397	0.104	0.313	0.229	0.076	0.241	0.397	0.396
7-Hexadecenal	Pearson Correlation	-0.015	0.219	**1.000** **	-0.060	-0.038	-0.096	-0.090	**-0.338** *	-0.193	**-0.464** **	**0.372** *	**0.394** *	-0.090	-0.091
	Sig. (2-tailed)	0.931	0.199	<0.001	0.730	0.828	0.577	0.600	0.044	0.260	0.004	0.025	0.018	0.600	0.599
11-Hexadecyn-1-ol	Pearson Correlation	0.146	-0.037	**0.818** **	-0.203	-0.091	-0.025	-0.216	-0.136	-0.175	**-0.433** **	0.326	**0.332** *	-0.098	-0.042
	Sig. (2-tailed)	0.395	0.831	<0.001	0.235	0.598	0.885	0.206	0.430	0.308	0.008	0.052	0.048	0.571	0.807
14-methyl-8-Hexadecenal	Pearson Correlation	0.168	-0.073	**0.836** **	-0.167	-0.133	-0.110	-0.178	-0.169	-0.206	**-0.334** *	0.306	0.276	-0.231	0.004
	Sig. (2-tailed)	0.327	0.672	<0.001	0.329	0.440	0.523	0.299	0.324	0.229	0.046	0.069	0.103	0.176	0.982
Methyl hexadecanoate	Pearson Correlation	**-0.355***	0.241	-0.091	**0.339** *	0.090	0.094	-0.091	0.047	0.071	**0.456** **	-0.107	**-0.354** *	-0.091	-0.091
	Sig. (2-tailed)	0.034	0.157	0.599	0.043	0.603	0.586	0.599	0.785	0.682	0.005	0.534	0.034	0.599	0.598
Hexadecanoic acid ethyl ester	Pearson Correlation	-0.187	0.327	**0.806** **	0.322	-0.016	0.186	-0.032	-0.276	-0.117	-0.118	0.290	0.112	-0.062	-0.003

	Sig. (2-tailed)	0.275	0.051	<0.001	0.056	0.928	0.277	0.851	0.103	0.496	0.492	0.086	0.517	0.720	0.987
Ethyl Oleate	Pearson Correlation	-0.300	**0.336***	**0.672****	0.241	-0.199	0.007	-0.166	-0.193	-0.155	0.046	0.189	-0.039	-0.166	0.113
	Sig. (2-tailed)	0.075	0.045	<0.001	0.156	0.245	0.969	0.332	0.258	0.366	0.790	0.269	0.823	0.332	0.511

LAB – lactic acid bacteria; TRIP – tryptamine; PHE – phenylethylamine; PUT – putrescine; CAD – cadaverine; HIS – histamine; TYR – tyramine; SPRMD – spermidine; SPRM – spermine; C14:0 - tetradecanoic acid; C16:0 - palmitic acid; C18:0 - stearic acid; C18:1 - 9-octadecenoic acid; C18:2 - linoleic acid; C18:3 α - α-linolenic acid; C20:0 - eicosanoic acid; C20:1 - cis-11-eicosenoic acid. ** Correlation is significant at the 0.01 level (2-tailed). * Correlation is significant at the 0.05 level (2-tailed). In **Bold** letters are marked significant values.

Table S11. Pearson correlations between volatiles and free amino acids (FAA) and γ-aminobutyric acid (GABA).

		Arginine	Glutamine	Asparagine	Glutamic acid	Serine	Aspartic acid	Threonine	Glycine	Alanine	GABA	Proline	Valine	Methionine	Phenylalanine	Leucine/Isoleucine	Lysine	Histidine
Hexanal	Pearson Correlation	0.562**	0.511**	0.077	-0.684**	0.308	-0.750**	-0.368*	-0.246	-0.305	0.178	-0.277	-0.453**	-0.379*	-0.551**	-0.559**	-0.339*	0.109
	Sig. (2-tailed)	<0.001	0.001	0.656	<0.001	0.067	<0.001	0.027	0.148	0.070	0.299	0.102	0.006	0.022	<0.001	<0.001	0.043	0.525
2-Hexenal	Pearson Correlation	0.534**	0.216	0.084	-0.719**	-0.007	-0.604**	-0.565**	-0.480**	-0.599**	-0.206	-0.499**	-0.585**	-0.211	-0.685**	-0.593**	-0.507**	-0.210
	Sig. (2-tailed)	<0.001	0.206	0.624	<0.001	0.969	<0.001	<0.001	0.003	<0.001	0.228	0.002	<0.001	0.217	<0.001	<0.001	0.002	0.219
1-Hexanol	Pearson Correlation	0.157	0.530**	0.074	-0.036	0.611**	-0.226	0.235	0.338*	0.273	0.338*	0.370*	0.164	-0.043	0.105	0.021	0.233	0.402*
	Sig. (2-tailed)	0.360	<0.001	0.670	0.833	<0.001	0.185	0.167	0.044	0.107	0.044	0.026	0.340	0.802	0.544	0.905	0.172	0.015
Hept-2-enal	Pearson Correlation	-0.321	0.037	-0.309	0.375*	0.334*	0.061	0.473**	0.532**	0.492**	0.577**	0.373*	0.468**	-0.008	0.364*	0.366*	0.299	0.079
	Sig. (2-tailed)	0.056	0.832	0.066	0.024	0.047	0.723	0.004	<0.001	0.002	<0.001	0.025	0.004	0.962	0.029	0.028	0.077	0.648
Benzaldehyde	Pearson Correlation	-0.446**	0.167	0.085	0.590**	0.756**	0.327	0.860**	0.740**	0.643**	0.476**	0.894**	0.883**	0.268	0.824**	0.813**	0.834**	0.209

	Sig. (2-tailed)	0.006	0.330	0.624	<0.001	<0.001	0.051	<0.001	<0.001	<0.001	0.003	<0.001	<0.001	0.113	<0.001	<0.001	<0.001	0.220
Vinyl amyl carbinol	Pearson Correlation	-0.324	0.235	0.240	0.355*	0.566**	0.230	0.621**	0.403*	0.414*	0.367*	0.686**	0.594**	-0.064	0.629**	0.547**	0.680**	0.238
	Sig. (2-tailed)	0.054	0.168	0.158	0.034	<0.001	0.177	<0.001	0.015	0.012	0.028	<0.001	<0.001	0.711	<0.001	<0.001	<0.001	0.162
Hexanoic acid	Pearson Correlation	-0.185	-0.156	-0.371*	0.245	0.022	0.051	0.193	0.313	0.289	0.298	0.247	0.228	0.053	0.239	0.209	0.147	0.088
	Sig. (2-tailed)	0.280	0.364	0.026	0.149	0.899	0.767	0.258	0.063	0.087	0.078	0.146	0.181	0.758	0.161	0.222	0.391	0.608
Acetic acid hexyl ester	Pearson Correlation	-0.373*	0.187	-0.083	0.541**	0.727**	0.193	0.803**	0.796**	0.688**	0.612**	0.807**	0.799**	0.242	0.700**	0.702**	0.722**	0.238
	Sig. (2-tailed)	0.025	0.275	0.632	<0.001	<0.001	0.258	<0.001	<0.001	<0.001	<0.001	<0.001	<0.001	0.155	<0.001	<0.001	<0.001	0.161
Oct-3-en-2-one	Pearson Correlation	-0.408*	0.080	0.168	0.544**	0.658**	0.357*	0.763**	0.645**	0.517**	0.228	0.802**	0.789**	0.370*	0.705**	0.737**	0.724**	0.110
	Sig. (2-tailed)	0.013	0.644	0.327	<0.001	<0.001	0.032	<0.001	<0.001	0.001	0.180	<0.001	<0.001	0.026	<0.001	<0.001	<0.001	0.522
Phenylacetaldehyde	Pearson Correlation	-0.461**	0.073	0.148	0.476**	0.438**	0.383*	0.663**	0.559**	0.462**	0.433**	0.565**	0.682**	0.123	0.569**	0.591**	0.510**	-0.025
	Sig. (2-tailed)	0.005	0.674	0.388	0.003	0.007	0.021	<0.001	<0.001	0.005	0.008	<0.001	<0.001	0.474	<0.001	<0.001	0.001	0.885
Oct-2-enal	Pearson Correlation	-0.469**	0.138	-0.082	0.428**	0.451**	0.219	0.584**	0.430**	0.450**	0.493**	0.515**	0.593**	-0.179	0.538**	0.487**	0.462**	0.080
	Sig. (2-tailed)	0.004	0.423	0.634	0.009	0.006	0.199	<0.001	0.009	0.006	0.002	0.001	<0.001	0.295	<0.001	0.003	0.005	0.642

Octahydro-2,3'-bifuran	Pearson Correlation	-0.217	0.228	0.080	0.130	**0.348***	-0.052	**0.353***	0.165	0.278	**0.390***	**0.399***	0.244	**-0.471****	0.283	0.161	**0.368***	0.246
	Sig. (2-tailed)	0.204	0.182	0.642	0.452	0.037	0.763	0.035	0.336	0.101	0.019	0.016	0.151	0.004	0.094	0.348	0.027	0.148
Nonanal	Pearson Correlation	**-0.488****	**-0.448****	-0.166	**0.526****	**-0.368***	**0.590****	0.236	0.151	0.166	-0.070	0.204	0.257	0.154	**0.354***	**0.372***	0.279	-0.119
	Sig. (2-tailed)	0.003	0.006	0.332	<0.001	0.027	<0.001	0.165	0.379	0.333	0.684	0.233	0.130	0.370	0.034	0.025	0.100	0.491
Phenethyl alcohol	Pearson Correlation	**-0.571****	-0.124	0.245	**0.719****	0.251	**0.721****	**0.743****	**0.596****	**0.447****	0.164	**0.626****	**0.720****	**0.449****	**0.660****	**0.767****	**0.734****	-0.073
	Sig. (2-tailed)	<0.001	0.472	0.150	<0.001	0.140	<0.001	<0.001	<0.001	0.006	0.339	<0.001	<0.001	0.006	<0.001	<0.001	<0.001	0.674
2-ethylhexanoic acid	Pearson Correlation	0.085	0.151	**0.409***	0.052	-0.037	0.124	0.152	0.209	0.316	0.247	0.045	0.064	0.227	0.083	0.051	0.173	0.272
	Sig. (2-tailed)	0.622	0.380	0.013	0.762	0.832	0.471	0.377	0.221	0.061	0.147	0.796	0.710	0.183	0.630	0.766	0.313	0.108
Non-2-enal	Pearson Correlation	**0.629****	**0.401***	0.175	**-0.795****	0.126	**-0.745****	**-0.512****	**-0.370***	**-0.423***	0.064	**-0.464****	**-0.550****	-0.287	**-0.655****	**-0.635****	**-0.501****	-0.015
	Sig. (2-tailed)	<0.001	0.015	0.308	<0.001	0.462	<0.001	0.001	0.026	0.010	0.712	0.004	<0.001	0.089	<0.001	<0.001	0.002	0.931
Octanoic acid	Pearson Correlation	-0.202	**-0.356***	0.084	0.143	**-0.552****	0.303	-0.071	-0.109	0.069	-0.037	-0.202	-0.046	-0.030	0.055	0.002	-0.120	-0.075
	Sig. (2-tailed)	0.238	0.033	0.627	0.404	<0.001	0.073	0.680	0.527	0.689	0.832	0.237	0.790	0.862	0.752	0.991	0.487	0.664
Decanal	Pearson Correlation	0.101	0.280	**0.476****	0.127	0.185	0.163	0.283	**0.354***	**0.423***	0.244	0.210	0.203	**0.392***	0.212	0.172	0.310	**0.377***

	Sig. (2-tailed)	0.558	0.098	0.003	0.461	0.281	0.343	0.095	0.034	0.010	0.152	0.220	0.234	0.018	0.215	0.317	0.066	0.024
Nona-2,4-dienal	Pearson Correlation	-0.317	0.056	0.033	**0.550****	**0.614****	**0.373***	**0.711****	**0.737****	**0.536****	0.294	**0.716****	**0.800****	**0.634****	**0.695****	**0.770****	**0.662****	0.080
	Sig. (2-tailed)	0.060	0.746	0.848	<0.001	<0.001	0.025	<0.001	<0.001	<0.001	0.082	<0.001	<0.001	<0.001	<0.001	<0.001	<0.001	0.641
Dec-2-enal	Pearson Correlation	-0.307	0.250	0.200	0.307	**0.535****	0.156	**0.576****	**0.359***	**0.400***	**0.410***	**0.640****	**0.524****	-0.192	**0.564****	**0.464****	**0.625****	0.258
	Sig. (2-tailed)	0.069	0.142	0.243	0.069	<0.001	0.364	<0.001	0.032	0.016	0.013	<0.001	0.001	0.263	<0.001	0.004	<0.001	0.128
Nonanoic acid	Pearson Correlation	0.109	**0.345***	**0.526****	0.113	0.217	0.117	**0.352***	**0.407***	**0.547****	**0.424****	0.318	0.226	0.260	0.281	0.173	**0.404***	**0.531****
	Sig. (2-tailed)	0.525	0.039	<0.001	0.510	0.204	0.495	0.036	0.014	<0.001	0.010	0.058	0.184	0.126	0.097	0.313	0.015	<0.001
Anethole	Pearson Correlation	0.069	-0.027	**0.682****	-0.051	-0.239	0.299	0.020	0.022	-0.015	-0.093	-0.105	-0.037	0.329	-0.077	-0.007	0.008	-0.097
	Sig. (2-tailed)	0.688	0.876	<0.001	0.769	0.160	0.076	0.910	0.900	0.931	0.591	0.544	0.831	0.050	0.656	0.966	0.962	0.575
Tridecane	Pearson Correlation	-0.310	-0.139	0.273	0.308	-0.040	**0.529****	0.256	0.168	0.085	-0.077	0.134	**0.354***	0.320	0.298	**0.360***	0.152	-0.239
	Sig. (2-tailed)	0.066	0.419	0.108	0.068	0.818	<0.001	0.132	0.328	0.622	0.657	0.437	0.034	0.057	0.078	0.031	0.376	0.161
Undecanal	Pearson Correlation	-0.309	**-0.473****	**-0.393***	0.257	-0.286	0.209	-0.001	-0.018	-0.077	-0.188	0.049	0.067	-0.038	0.103	0.135	-0.024	-0.243
	Sig. (2-tailed)	0.066	0.004	0.018	0.130	0.091	0.222	0.995	0.916	0.653	0.272	0.775	0.699	0.826	0.550	0.432	0.890	0.154

2-methylpropanoic acid 2,2-dimethyl-1-(2-hydroxy-1-methylethyl)propyl ester	Pearson Correlation	-0.073	-0.057	0.156	0.090	-0.201	0.158	0.008	-0.046	0.055	-0.044	-0.123	-0.039	0.047	-0.013	-0.001	0.028	0.015
	Sig. (2-tailed)	0.671	0.742	0.362	0.600	0.240	0.357	0.963	0.791	0.751	0.799	0.476	0.823	0.784	0.942	0.996	0.871	0.929
Eugenol	Pearson Correlation	0.206	0.120	**0.728****	-0.036	-0.099	0.215	0.101	0.175	0.203	0.052	0.050	0.010	**0.430****	0.037	0.031	0.166	0.230
	Sig. (2-tailed)	0.227	0.487	<0.001	0.835	0.566	0.207	0.558	0.307	0.234	0.763	0.770	0.952	0.009	0.828	0.859	0.332	0.176
2-methylpropanoic acid 2-ethyl-3-hydroxyhexyl ester	Pearson Correlation	-0.157	**-0.333***	0.128	0.161	**-0.496****	0.292	-0.051	-0.089	0.001	-0.167	-0.140	-0.110	0.048	-0.028	-0.014	-0.001	-0.061
	Sig. (2-tailed)	0.362	0.047	0.457	0.349	0.002	0.084	0.768	0.606	0.995	0.331	0.414	0.521	0.782	0.872	0.934	0.996	0.722
β-Damascenone	Pearson Correlation	**-0.345***	**-0.610****	**-0.409***	0.226	**-0.599****	0.300	-0.182	-0.243	-0.241	**-0.356***	-0.180	-0.113	-0.132	-0.030	0.004	-0.189	**-0.361***
	Sig. (2-tailed)	0.039	<0.001	0.013	0.185	<0.001	0.076	0.287	0.154	0.157	0.033	0.293	0.512	0.441	0.862	0.982	0.271	0.030
1-Tetradecene	Pearson Correlation	**0.502****	**0.422***	0.045	**-0.629****	0.111	**-0.5**	**-0.404***	-0.300	**-0.335***	0.101	**-0.341***	**-0.4**	-0.229	**-0.520****	**-0.507****	**-0.348***	0.018

							**51****						**55****					
	Sig. (2-tailed)	0.002	0.010	0.795	<0.001	0.518	<0.001	0.014	0.075	0.046	0.557	0.042	0.005	0.180	0.001	0.002	0.037	0.916
Tetradecane	Pearson Correlation	**-0.469****	**-0.654****	-0.211	**0.439****	**-0.554****	**0.562****	0.047	-0.059	-0.097	**-0.345***	-0.005	0.102	0.134	0.180	0.264	0.081	**-0.359***
	Sig. (2-tailed)	0.004	<0.001	0.217	0.007	<0.001	<0.001	0.787	0.731	0.573	0.039	0.978	0.552	0.436	0.294	0.120	0.641	0.031
Dodecanal	Pearson Correlation	0.084	-0.069	0.064	0.061	-0.297	0.126	-0.030	0.126	0.253	0.094	0.014	-0.077	0.178	0.034	-0.069	-0.008	0.295
	Sig. (2-tailed)	0.625	0.688	0.710	0.726	0.078	0.463	0.860	0.464	0.137	0.587	0.934	0.657	0.299	0.846	0.689	0.963	0.081
3-(2,2,6-Trimethyl-7-oxabicyclo[4.1.0]heptan-1-yl)prop-2-enal	Pearson Correlation	**-0.347***	**-0.550****	-0.277	0.306	**-0.561****	**0.377***	-0.058	-0.106	-0.085	-0.252	-0.072	-0.040	-0.039	0.057	0.079	-0.039	-0.230
	Sig. (2-tailed)	0.038	<0.001	0.102	0.069	<0.001	0.024	0.738	0.539	0.621	0.139	0.677	0.818	0.819	0.739	0.649	0.822	0.177
Tridecanal	Pearson Correlation	0.177	-0.084	-0.286	-0.287	-0.052	**-0.429****	**-0.409***	**-0.413***	**-0.491****	-0.312	**-0.349***	**-0.404***	**-0.428****	**-0.410***	**-0.378***	**-0.358***	-0.173
	Sig. (2-tailed)	0.302	0.625	0.091	0.090	0.765	0.009	0.013	0.012	0.002	0.064	0.037	0.015	0.009	0.013	0.023	0.032	0.314
((Butoxymethylethoxy)m	Pearson Correlation	-0.056	-0.246	0.170	0.141	**-0.402***	0.272	0.010	0.066	0.100	-0.029	-0.034	-0.061	0.199	0.003	0.022	0.072	0.031

ethyleth oxy)-propanol	Sig. (2-tailed)	0.744	0.149	0.320	0.411	0.015	0.108	0.953	0.702	0.561	0.868	0.846	0.722	0.244	0.984	0.900	0.677	0.856
Hexadecane	Pearson Correlation	-0.606**	-0.644**	-0.511**	0.445**	-0.343*	0.342*	0.111	-0.016	-0.026	-0.149	0.073	0.222	-0.215	0.243	0.272	-0.011	-0.394*
	Sig. (2-tailed)	<0.001	<0.001	0.001	0.007	0.040	0.041	0.520	0.928	0.881	0.386	0.674	0.194	0.208	0.153	0.109	0.948	0.017
Tetradecanal	Pearson Correlation	0.196	-0.029	-0.098	-0.211	-0.054	-0.177	-0.307	-0.310	-0.228	-0.330*	-0.257	-0.164	0.105	-0.106	-0.126	-0.244	-0.026
	Sig. (2-tailed)	0.251	0.866	0.568	0.217	0.754	0.302	0.069	0.066	0.181	0.049	0.130	0.340	0.544	0.539	0.463	0.152	0.882
Tridecanedial	Pearson Correlation	-0.125	0.024	0.216	0.157	0.129	0.348*	0.145	0.084	-0.050	-0.165	0.109	0.257	0.340*	0.208	0.274	0.125	-0.183
	Sig. (2-tailed)	0.469	0.889	0.206	0.360	0.453	0.037	0.399	0.627	0.772	0.335	0.527	0.130	0.042	0.224	0.106	0.469	0.285
Pentadecanal	Pearson Correlation	-0.135	-0.289	-0.330*	0.061	-0.188	0.063	-0.199	-0.300	-0.235	-0.382*	-0.227	-0.017	0.028	0.037	0.058	-0.190	-0.246
	Sig. (2-tailed)	0.431	0.087	0.049	0.723	0.272	0.716	0.244	0.075	0.168	0.021	0.183	0.920	0.873	0.829	0.739	0.266	0.149
cis,cis-7,10,-Hexadecadienal	Pearson Correlation	-0.273	-0.416*	-0.292	0.208	-0.164	0.185	-0.042	-0.171	-0.155	-0.312	-0.093	0.156	0.096	0.205	0.253	-0.036	-0.311
	Sig. (2-tailed)	0.107	0.012	0.085	0.223	0.339	0.281	0.806	0.318	0.367	0.064	0.590	0.364	0.576	0.230	0.137	0.834	0.065
7-Hexadecenal	Pearson Correlation	-0.214	-0.481**	-0.405*	0.069	-0.410*	0.046	-0.250	-0.343*	-0.237	-0.329	-0.342*	-0.097	-0.112	-0.046	-0.016	-0.298	-0.339*
	Sig. (2-tailed)	0.211	0.003	0.014	0.690	0.013	0.791	0.142	0.041	0.164	0.050	0.041	0.575	0.516	0.792	0.925	0.077	0.043

11-Hexadecyn-1-ol	Pearson Correlation	-0.122	-0.236	**-0.490****	0.074	-0.173	0.017	-0.244	-0.315	-0.238	**-0.415***	-0.256	-0.081	-0.025	-0.025	-0.020	-0.245	-0.192
	Sig. (2-tailed)	0.480	0.166	0.002	0.669	0.314	0.923	0.152	0.061	0.162	0.012	0.131	0.639	0.884	0.883	0.908	0.150	0.261
14-methyl-8-Hexadecenal	Pearson Correlation	-0.059	-0.198	**-0.341***	-0.009	-0.194	0.016	-0.271	**-0.349***	-0.258	**-0.415***	-0.282	-0.108	0.010	-0.049	-0.048	-0.259	-0.188
	Sig. (2-tailed)	0.732	0.247	0.042	0.961	0.257	0.928	0.110	0.037	0.129	0.012	0.096	0.530	0.955	0.779	0.781	0.128	0.271
Methyl hexadecanoate	Pearson Correlation	-0.214	0.076	0.255	**0.388***	**0.431****	**0.431****	**0.510****	**0.428****	0.297	0.087	**0.551****	**0.620****	**0.543****	**0.626****	**0.656****	**0.588****	0.059
	Sig. (2-tailed)	0.210	0.661	0.133	0.019	0.009	0.009	0.001	0.009	0.078	0.613	<0.001	<0.001	<0.001	<0.001	<0.001	<0.001	0.733
Hexadecanoic acid ethyl ester	Pearson Correlation	-0.311	-0.292	-0.127	**0.362***	-0.153	**0.411***	0.108	-0.002	0.074	-0.284	0.025	0.258	0.318	0.316	**0.338***	0.106	-0.134
	Sig. (2-tailed)	0.065	0.084	0.462	0.030	0.373	0.013	0.529	0.989	0.670	0.093	0.887	0.128	0.059	0.060	0.044	0.537	0.437
Ethyl Oleate	Pearson Correlation	**-0.362***	-0.309	-0.092	**0.345***	0.051	**0.334***	0.214	0.026	0.020	-0.220	0.190	**0.393***	0.214	**0.437****	**0.473****	0.237	-0.216
	Sig. (2-tailed)	0.030	0.067	0.594	0.039	0.769	0.047	0.211	0.878	0.910	0.197	0.266	0.018	0.210	0.008	0.004	0.164	0.205

GABA – γ-aminobutyric acid. ** Correlation is significant at the 0.01 level (2-tailed). * Correlation is significant at the 0.05 level (2-tailed). In **Bold** letters are marked significant values.

Table S12. Significance of analysed factors (different growing conditions of lentils, different fermentation conditions, and different duration of fermentation) and their interaction in volatile compound (VC) lentil wholemeal composition.

Factor	Dependent variable	p
Growing conditions	Hexanal	**<0.001**
	2-Hexenal	**<0.001**
	1-Hexanol	**<0.001**
	Hept-2-enal	**<0.001**
	Benzaldehyde	**<0.001**
	Vinyl amyl carbinol	**<0.001**
	Hexanoic acid	**<0.001**
	Acetic acid hexyl ester	**<0.001**
	Oct-3-en-2-one	**<0.001**
	Phenylacetaldehyde	**<0.001**
	Oct-2-enal	0.345
	Octahydro-2,3'-bifuran	**<0.001**
	Nonanal	**<0.001**
	Phenethyl alcohol	**<0.001**
	2-ethylhexanoic acid	**<0.001**
	Non-2-enal	**<0.001**
	Octanoic acid	0.640
	Decanal	**<0.001**
	Nona-2,4-dienal	0.457
	Dec-2-enal	**<0.001**
	Nonanoic acid	**<0.001**
	Anethole	**<0.001**
	Tridecane	**<0.001**
	Undecanal	**<0.001**
	2-methylpropanoic acid 2,2-dimethyl-1-(2-hydroxy-1-methylethyl)propyl ester	**<0.001**
	Eugenol	**<0.001**
	2-methylpropanoic acid 2-ethyl-3-hydroxyhexyl ester	**0.009**
	β-Damascenone	**<0.001**
	1-Tetradecene	**<0.001**
	Tetradecane	**<0.001**
	Dodecanal	**<0.001**
	3-(2,2,6-Trimethyl-7-oxabicyclo[4.1.0]heptan-1-yl)prop-2-enal	**<0.001**
	Tridecanal	**<0.001**
	((Butoxymethylethoxy)methylethoxy)-propanol	0.632
	Hexadecane	**<0.001**
	Tetradecanal	**<0.001**
	Tridecanedial	**<0.001**
	Pentadecanal	**<0.001**
	cis,cis-7,10,-Hexadecadienal	**<0.001**

	7-Hexadecenal	**<0.001**
	11-Hexadecyn-1-ol	**<0.001**
	14-methyl-8-Hexadecenal	**<0.001**
	Methyl hexadecanoate	**<0.001**
	Hexadecanoic acid ethyl ester	**0.006**
	Ethyl Oleate	**<0.001**
Type of fermentation	Hexanal	**<0.001**
	2-Hexenal	**<0.001**
	1-Hexanol	**<0.001**
	Hept-2-enal	**<0.001**
	Benzaldehyde	**<0.001**
	Vinyl amyl carbinol	**<0.001**
	Hexanoic acid	**0.002**
	Acetic acid hexyl ester	**<0.001**
	Oct-3-en-2-one	**<0.001**
	Phenylacetaldehyde	**<0.001**
	Oct-2-enal	**<0.001**
	Octahydro-2,3'-bifuran	**<0.001**
	Nonanal	**<0.001**
	Phenethyl alcohol	**<0.001**
	2-ethylhexanoic acid	**<0.001**
	Non-2-enal	**<0.001**
	Octanoic acid	**<0.001**
	Decanal	0.483
	Nona-2,4-dienal	**<0.001**
	Dec-2-enal	**<0.001**
	Nonanoic acid	**0.008**
	Anethole	**<0.001**
	Tridecane	**<0.001**
	Undecanal	**<0.001**
	2-methylpropanoic acid 2,2-dimethyl-1-(2-hydroxy-1-methylethyl)propyl ester	**<0.001**
	Eugenol	**<0.001**
	2-methylpropanoic acid 2-ethyl-3-hydroxyhexyl ester	**<0.001**
	β-Damascenone	**<0.001**
	1-Tetradecene	**<0.001**
	Tetradecane	**<0.001**
	Dodecanal	**<0.001**
	3-(2,2,6-Trimethyl-7-oxabicyclo[4.1.0]heptan-1-yl)prop-2-enal	**<0.001**
	Tridecanal	**<0.001**
	((Butoxymethylethoxy)methylethoxy)-propanol	**<0.001**
	Hexadecane	**<0.001**
	Tetradecanal	**<0.001**
	Tridecanedial	**<0.001**
	Pentadecanal	**<0.001**

	cis,cis-7,10,-Hexadecadienal	**<0.001**
	7-Hexadecenal	**<0.001**
	11-Hexadecyn-1-ol	**<0.001**
	14-methyl-8-Hexadecenal	**<0.001**
	Methyl hexadecanoate	**<0.001**
	Hexadecanoic acid ethyl ester	**<0.001**
	Ethyl Oleate	**<0.001**
Fermentation duration	Hexanal	**<0.001**
	2-Hexenal	**<0.001**
	1-Hexanol	0.146
	Hept-2-enal	**0.002**
	Benzaldehyde	**<0.001**
	Vinyl amyl carbinol	**<0.001**
	Hexanoic acid	**<0.001**
	Acetic acid hexyl ester	**<0.001**
	Oct-3-en-2-one	**<0.001**
	Phenylacetaldehyde	**<0.001**
	Oct-2-enal	**<0.001**
	Octahydro-2,3'-bifuran	**<0.001**
	Nonanal	**<0.001**
	Phenethyl alcohol	**<0.001**
	2-ethylhexanoic acid	**<0.001**
	Non-2-enal	**<0.001**
	Octanoic acid	**<0.001**
	Decanal	**<0.001**
	Nona-2,4-dienal	**<0.001**
	Dec-2-enal	**<0.001**
	Nonanoic acid	**<0.001**
	Anethole	0.755
	Tridecane	**<0.001**
	Undecanal	**<0.001**
	2-methylpropanoic acid 2,2-dimethyl-1-(2-hydroxy-1-methylethyl)propyl ester	**<0.001**
	Eugenol	**<0.001**
	2-methylpropanoic acid 2-ethyl-3-hydroxyhexyl ester	**0.024**
	β-Damascenone	**<0.001**
	1-Tetradecene	**<0.001**
	Tetradecane	**<0.001**
	Dodecanal	**<0.001**
	3-(2,2,6-Trimethyl-7-oxabicyclo[4.1.0]heptan-1-yl)prop-2-enal	**<0.001**
	Tridecanal	**<0.001**
	((Butoxymethylethoxy)methylethoxy)-propanol	**<0.001**
	Hexadecane	**0.003**
	Tetradecanal	**<0.001**
	Tridecanedial	**<0.001**

	Pentadecanal	**<0.001**
	cis,cis-7,10,-Hexadecadienal	**<0.001**
	7-Hexadecenal	**<0.001**
	11-Hexadecyn-1-ol	**<0.001**
	14-methyl-8-Hexadecenal	**<0.001**
	Methyl hexadecanoate	**<0.001**
	Hexadecanoic acid ethyl ester	**<0.001**
	Ethyl Oleate	**<0.001**
Growing conditions * Type of fermentation	Hexanal	**<0.001**
	2-Hexenal	**<0.001**
	1-Hexanol	**<0.001**
	Hept-2-enal	**<0.001**
	Benzaldehyde	**<0.001**
	Vinyl amyl carbinol	**<0.001**
	Hexanoic acid	0.514
	Acetic acid hexyl ester	**<0.001**
	Oct-3-en-2-one	**<0.001**
	Phenylacetaldehyde	**<0.001**
	Oct-2-enal	**<0.001**
	Octahydro-2,3'-bifuran	**<0.001**
	Nonanal	0.140
	Phenethyl alcohol	**<0.001**
	2-ethylhexanoic acid	**<0.001**
	Non-2-enal	**<0.001**
	Octanoic acid	0.051
	Decanal	**<0.001**
	Nona-2,4-dienal	0.457
	Dec-2-enal	**<0.001**
	Nonanoic acid	**<0.001**
	Anethole	**<0.001**
	Tridecane	**<0.001**
	Undecanal	**<0.001**
	2-methylpropanoic acid 2,2-dimethyl-1-(2-hydroxy-1-methylethyl)propyl ester	**<0.001**
	Eugenol	**<0.001**
	2-methylpropanoic acid 2-ethyl-3-hydroxyhexyl ester	**<0.001**
	β-Damascenone	**<0.001**
	1-Tetradecene	**<0.001**
	Tetradecane	**<0.001**
	Dodecanal	0.372
	3-(2,2,6-Trimethyl-7-oxabicyclo[4.1.0]heptan-1-yl)prop-2-enal	**<0.001**
	Tridecanal	**<0.001**
	((Butoxymethylethoxy)methylethoxy)-propanol	**<0.001**
	Hexadecane	**<0.001**
	Tetradecanal	**<0.001**

	Tridecanedial	**<0.001**
	Pentadecanal	**<0.001**
	cis,cis-7,10,-Hexadecadienal	**<0.001**
	7-Hexadecenal	**<0.001**
	11-Hexadecyn-1-ol	**<0.001**
	14-methyl-8-Hexadecenal	**<0.001**
	Methyl hexadecanoate	**<0.001**
	Hexadecanoic acid ethyl ester	**<0.001**
	Ethyl Oleate	**<0.001**
Growing conditions * Fermentation duration	Hexanal	**<0.001**
	2-Hexenal	**<0.001**
	1-Hexanol	**<0.001**
	Hept-2-enal	**<0.001**
	Benzaldehyde	0.108
	Vinyl amyl carbinol	**<0.001**
	Hexanoic acid	**<0.001**
	Acetic acid hexyl ester	**<0.001**
	Oct-3-en-2-one	**<0.001**
	Phenylacetaldehyde	**<0.001**
	Oct-2-enal	**0.004**
	Octahydro-2,3'-bifuran	**<0.001**
	Nonanal	0.983
	Phenethyl alcohol	**<0.001**
	2-ethylhexanoic acid	**<0.001**
	Non-2-enal	0.279
	Octanoic acid	**0.002**
	Decanal	**<0.001**
	Nona-2,4-dienal	0.457
	Dec-2-enal	**<0.001**
	Nonanoic acid	**<0.001**
	Anethole	0.755
	Tridecane	**<0.001**
	Undecanal	**<0.001**
	2-methylpropanoic acid 2,2-dimethyl-1-(2-hydroxy-1-methylethyl)propyl ester	**<0.001**
	Eugenol	**<0.001**
	2-methylpropanoic acid 2-ethyl-3-hydroxyhexyl ester	**<0.001**
	β-Damascenone	**<0.001**
	1-Tetradecene	**<0.001**
	Tetradecane	**0.012**
	Dodecanal	**<0.001**
	3-(2,2,6-Trimethyl-7-oxabicyclo[4.1.0]heptan-1-yl)prop-2-enal	**<0.001**
	Tridecanal	**<0.001**
	((Butoxymethylethoxy)methylethoxy)-propanol	0.396
	Hexadecane	**<0.001**

	Tetradecanal	**<0.001**
	Tridecanedial	**<0.001**
	Pentadecanal	**<0.001**
	cis,cis-7,10,-Hexadecadienal	**<0.001**
	7-Hexadecenal	**<0.001**
	11-Hexadecyn-1-ol	**<0.001**
	14-methyl-8-Hexadecenal	**<0.001**
	Methyl hexadecanoate	**<0.001**
	Hexadecanoic acid ethyl ester	**<0.001**
	Ethyl Oleate	**<0.001**
Type of fermentation * Fermentation duration	Hexanal	**<0.001**
	2-Hexenal	**<0.001**
	1-Hexanol	0.081
	Hept-2-enal	**<0.001**
	Benzaldehyde	**<0.001**
	Vinyl amyl carbinol	**<0.001**
	Hexanoic acid	**<0.001**
	Acetic acid hexyl ester	**<0.001**
	Oct-3-en-2-one	**<0.001**
	Phenylacetaldehyde	**<0.001**
	Oct-2-enal	**<0.001**
	Octahydro-2,3'-bifuran	**<0.001**
	Nonanal	**<0.001**
	Phenethyl alcohol	**0.010**
	2-ethylhexanoic acid	0.623
	Non-2-enal	**<0.001**
	Octanoic acid	0.189
	Decanal	**<0.001**
	Nona-2,4-dienal	**<0.001**
	Dec-2-enal	**<0.001**
	Nonanoic acid	**<0.001**
	Anethole	**<0.001**
	Tridecane	**<0.001**
	Undecanal	**<0.001**
	2-methylpropanoic acid 2,2-dimethyl-1-(2-hydroxy-1-methylethyl)propyl ester	0.989
	Eugenol	**<0.001**
	2-methylpropanoic acid 2-ethyl-3-hydroxyhexyl ester	**<0.001**
	β-Damascenone	**<0.001**
	1-Tetradecene	**<0.001**
	Tetradecane	**<0.001**
	Dodecanal	**<0.001**
	3-(2,2,6-Trimethyl-7-oxabicyclo[4.1.0]heptan-1-yl)prop-2-enal	**<0.001**
	Tridecanal	**<0.001**
	((Butoxymethylethoxy)methylethoxy)-propanol	**<0.001**

	Hexadecane	0.075
	Tetradecanal	**<0.001**
	Tridecanedial	**<0.001**
	Pentadecanal	**<0.001**
	cis,cis-7,10,-Hexadecadienal	**<0.001**
	7-Hexadecenal	**<0.001**
	11-Hexadecyn-1-ol	**<0.001**
	14-methyl-8-Hexadecenal	**<0.001**
	Methyl hexadecanoate	**<0.001**
	Hexadecanoic acid ethyl ester	**<0.001**
	Ethyl Oleate	**<0.001**
Growing conditions * Type of fermentation * Fermentation duration	Hexanal	**<0.001**
	2-Hexenal	**<0.001**
	1-Hexanol	**<0.001**
	Hept-2-enal	**0.017**
	Benzaldehyde	**<0.001**
	Vinyl amyl carbinol	**<0.001**
	Hexanoic acid	**<0.001**
	Acetic acid hexyl ester	**<0.001**
	Oct-3-en-2-one	**<0.001**
	Phenylacetaldehyde	**<0.001**
	Oct-2-enal	**<0.001**
	Octahydro-2,3'-bifuran	**<0.001**
	Nonanal	0.050
	Phenethyl alcohol	**<0.001**
	2-ethylhexanoic acid	**<0.001**
	Non-2-enal	**<0.001**
	Octanoic acid	0.146
	Decanal	**<0.001**
	Nona-2,4-dienal	0.457
	Dec-2-enal	**<0.001**
	Nonanoic acid	**<0.001**
	Anethole	**<0.001**
	Tridecane	**<0.001**
	Undecanal	**<0.001**
	2-methylpropanoic acid 2,2-dimethyl-1-(2-hydroxy-1-methylethyl)propyl ester	**<0.001**
	Eugenol	**<0.001**
	2-methylpropanoic acid 2-ethyl-3-hydroxyhexyl ester	**<0.001**
	β-Damascenone	**<0.001**
	1-Tetradecene	**<0.001**
	Tetradecane	**0.002**
	Dodecanal	**<0.001**
	3-(2,2,6-Trimethyl-7-oxabicyclo[4.1.0]heptan-1-yl)prop-2-enal	**<0.001**
	Tridecanal	**<0.001**

	((Butoxymethylethoxy)methylethoxy)-propanol	**<0.001**
	Hexadecane	**<0.001**
	Tetradecanal	**<0.001**
	Tridecanedial	**<0.001**
	Pentadecanal	**<0.001**
	cis,cis-7,10,-Hexadecadienal	**<0.001**
	7-Hexadecenal	**<0.001**
	11-Hexadecyn-1-ol	**<0.001**
	14-methyl-8-Hexadecenal	**<0.001**
	Methyl hexadecanoate	**<0.001**
	Hexadecanoic acid ethyl ester	**<0.001**
	Ethyl Oleate	**<0.001**

p – significance. The influence of analysed factors and their interaction are significant when p ≤ 0.05. In **Bold** letters are marked significant values.

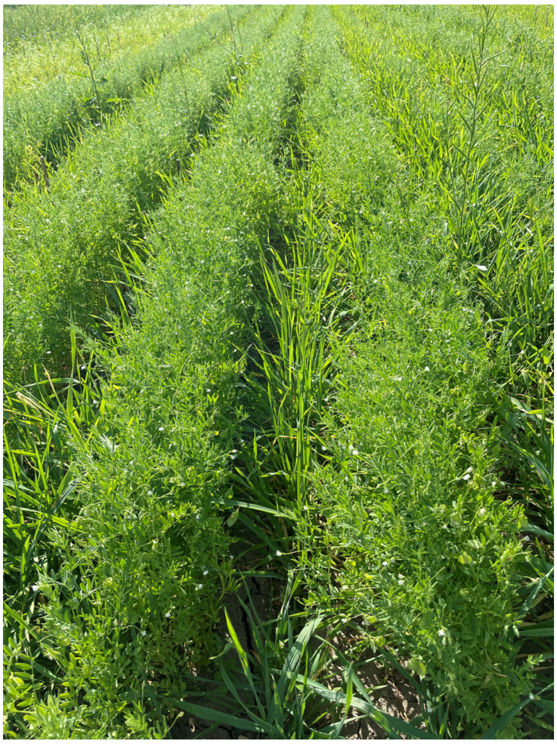

Figure S1. Image of the relay intercropped lentil with winter rye (LR).
